# Extracellular vesicles in cancers: mechanisms, biomarkers, and therapeutic strategies

**DOI:** 10.1002/mco2.70009

**Published:** 2024-11-28

**Authors:** Yuxi Ma, Xiaohui Zhang, Cuiwei Liu, Yanxia Zhao

**Affiliations:** ^1^ Cancer Center Union Hospital Tongji Medical College Huazhong University of Science and Technology Wuhan China; ^2^ Hubei Key Laboratory of Precision Radiation Oncology Wuhan China; ^3^ Cancer Center Institute of Radiation Oncology Union Hospital Tongji Medical College Huazhong University of Science and Technology Wuhan China; ^4^ Cancer Center Hubei Key Laboratory of Cell Homeostasis College of Life Sciences TaiKang Center for Life and Medical Sciences Wuhan University Wuhan China

**Keywords:** biomarkers, cancer, cargo, challenges, delivery vehicles, exosomes, extracellular vesicles, therapy resistance, tumor microenvironment

## Abstract

Extracellular vesicles (EVs) composed of various biologically active constituents, such as proteins, nucleic acids, lipids, and metabolites, have emerged as a noteworthy mode of intercellular communication. There are several categories of EVs, including exosomes, microvesicles, and apoptotic bodies, which largely differ in their mechanisms of formation and secretion. The amount of evidence indicated that changes in the EV quantity and composition play a role in multiple aspects of cancer development, such as the transfer of oncogenic signals, angiogenesis, metabolism remodeling, and immunosuppressive effects. As EV isolation technology and characteristics recognition improve, EVs are becoming more commonly used in the early diagnosis and evaluation of treatment effectiveness for cancers. Actually, EVs have sparked clinical interest in their potential use as delivery vehicles or vaccines for innovative antitumor techniques. This review will focus on the function of biological molecules contained in EVs linked to cancer progression and their participation in the intricate interrelationship within the tumor microenvironment. Furthermore, the potential efficacy of an EV‐based liquid biopsy and delivery cargo for treatment will be explored. Finally, we explicitly delineate the limitations of EV‐based anticancer therapies and provide an overview of the clinical trials aimed at improving EV development.

## Graphical abstract

Extracellular vesicles (EVs) composed of proteins, nucleic acids, lipids, and metabolites are a notable mechanism of intercellular communication. This review will examine how biological components in EVs affect cancer growth and the tumor microenvironment's complex interactions. We will also test EV‐based liquid biopsy and delivery cargo for therapy. Finally, we outline the current trials to improve EV development.

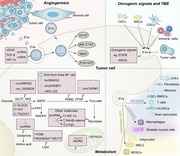

## 1 INTRODUCTION

Extracellular vesicles (EVs) released by different types of cells are crucial for their communication in the tumor microenvironment (TME). Studies have demonstrated that EVs are capable of delivering various biomolecules, such as proteins, nucleic acids, lipids, and metabolites, and eliciting biological alterations in the target cells.^1^ Emerging evidence points to EVs as an essential biological entity contributing to maintaining cell growth signaling, promoting invasion and metastasis, stimulating angiogenesis, disrupting cellular metabolism, and evading the immune system.^2^, ^3^

In recent years, a burgeoning body of evidence has emerged to substantiate the involvement of EVs in transferring the oncogenic molecules to regulate several signaling pathways, such as the epidermal growth factor receptor (EGFR) and Kirsten rat sarcoma virus (KRAS) signaling pathways, the PI3K/AKT, and the MAPK/ERK pathways.^4^ Consequently, cell differentiation, aggressive phenotype, proliferation and therapy resistance can be influenced by these pathways.^5^ Additionally, recent studies have identified the essential contributions of EVs to angiogenesis, which is typically induced by soluble proangiogenic factors, such as vascular endothelial growth factor (VEGF) secreted by cancer cells in hypoxic conditions.^6^ Targeting EVs has emerged as a viable strategy to manipulate angiogenesis for cancer treatment.

The TME is comprised of a complex network of cells and structures that envelop neoplastic cells. The major constituents of the TME are immune cells and immunosuppressive molecules, such as cytokines, growth factors, and immune checkpoint inhibitors (ICIs). The development of cancer and the response to immunotherapy is significantly influenced by the immunosuppressive microenvironment mediated by EVs.^7^ Although EVs from immune cells can facilitate metastasis and promote cancer growth by transferring PD‐L1, reducing the cytotoxicity of cytotoxic T lymphocytes (CTLs), and creating an immunosuppressive TME, they can also generate anticancer immune responses through the delivery of tumor suppressors or tumor antigens.^8^, ^9^ The application of the immunomodulatory capabilities of EVs is an important therapeutic approach for overcoming resistance to immune therapy. It has been widely acknowledged that alteration in cellular metabolism is a pivotal occurrence in cancer. Numerous modifications in metabolic pathways, including amino acid, nucleotide biosynthesis, fatty acid metabolism, and glucose metabolism, have been documented.^10^ These mechanisms have primarily centered around EVs.^11^

The transportation of EV cargo to malignant cells is linked to the development of resistance to tumor therapy, including targeted therapy, chemotherapy, radiotherapy, and ICI therapy.^12^ Overall, understanding the molecular mechanisms will facilitate the development of novel approaches and drugs that specifically target EV‐mediated carcinogenesis. Thus, many pharmacological agents are being explored with regard to lipid membrane, cytoskeleton structure, biogenesis, secretion, and recipient cell uptake.^13^ Actually, EVs have garnered significant attention as vehicles for delivering various therapeutic compounds such as RNAs, proteins, and synthetic medicines. Translational medicine has shown interest in using EVs found in body fluids, including blood, milk, urine, saliva, and cerebrospinal fluid, as a source for liquid biopsies for early cancer diagnosis, assess disease status, and therapy response.^14^ The primary advantage of liquid biopsy lies in its capacity to offer pathological insights before and throughout treatment, hence facilitating personalized cancer therapy.^15^ Intriguingly, certain studies have discovered that EVs can also impede tumor progression. This phenomenon is mediated by engineered EVs or by antigen presentation to CTLs such as dendritic cell (DC)‐derived exosomes (Dex).^16^

The purpose of this review is to conduct a comprehensive analysis of the biological properties of EVs and their role as intercellular messengers in the TME, constructing an intricate web of signaling relationships that carry out pro‐ and antitumor effects. Moreover, we summarized the strong correlation between EVs and the development of angiogenesis, oncogene transfer, immune suppression, metabolism abnormalities and treatment resistance in tumors. Additionally, we explore the function of EVs as biomarkers for tumor diagnostics and prognosis and as drug carriers for cancer therapy. Translation of EVs from the laboratory to the clinic necessitates surmounting numerous obstacles. Although facing these obstacles, EVs have demonstrated significant promise in clinical settings and will propel the progress of precision cancer medicine.

## 2 BIOGENESIS AND COMPOSITION OF EVs

### 2.1 Types of EVs: exosomes, microvesicles, and apoptotic bodies

**FIGURE 1:**
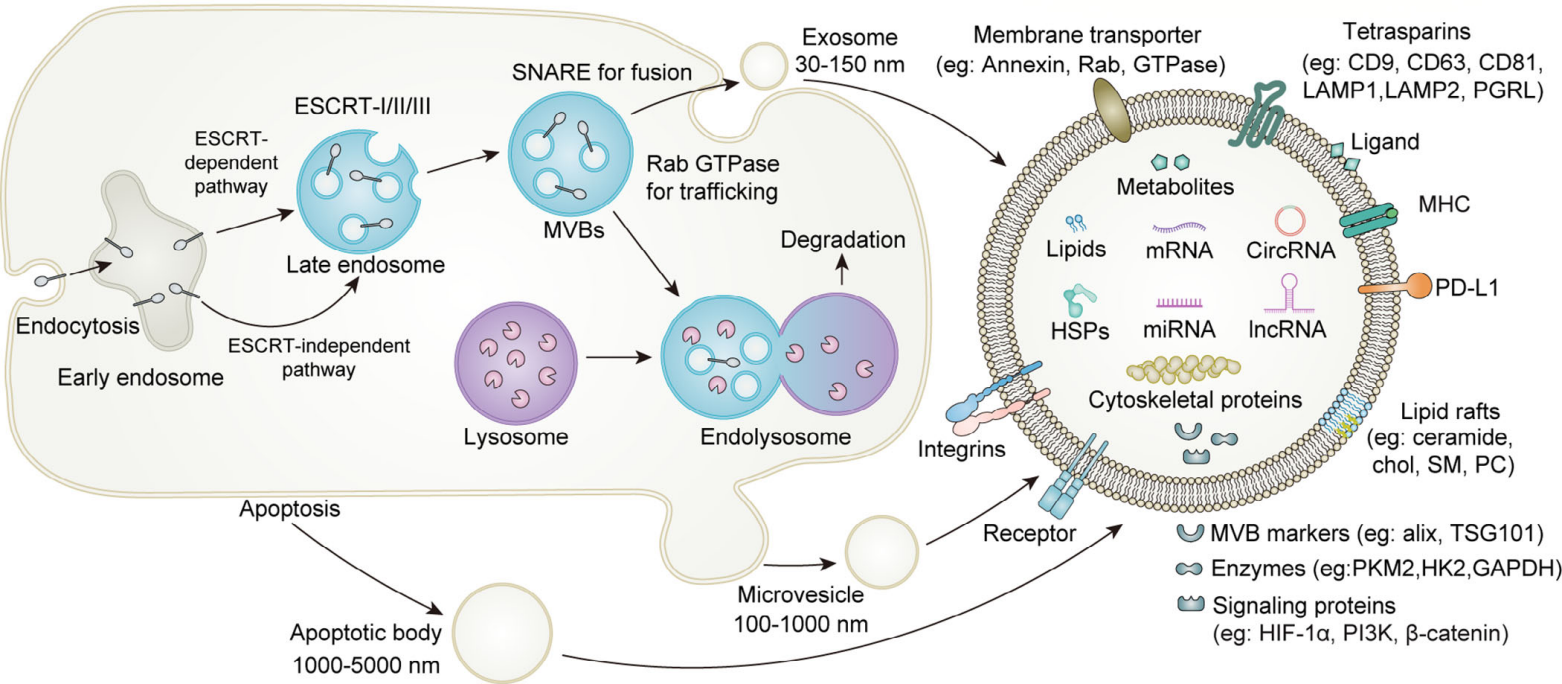
Biogenesis and components of EVs. The cell membrane protrudes inward, resulting in the formation of early endosomes. After maturation into late endosomes, MVBs are subsequently initiated. Alternatively, late MVBs fuse with lysosomes for degradation or release exosomes by fusing with the plasma membrane. Microvesicles biogenesis occur directly through budding from the plasma membrane and apoptotic bodies are released during apoptosis. Upon release from cells, EVs transport a variety of biological constituents to perform specific functions, including an array of proteins (membrane proteins, signaling proteins, enzymes, markers, etc.), RNA species, DNA, lipids, and metabolites. EVs, extracellular vesicles; MVBs, multivesicular bodies.

In the 1980s, Pan and Johnstone demonstrated that peptide‐containing vesicles are discharged into the extracellular space from sheep reticulocytes. Since then, the term “exosome” has been applied to EVs.^17^ Depending on their size and biogenesis, the taxonomy of EVs typically includes three types of vesicles: exosomes (<150 nm in diameter), microvesicles (MVs) (100–1000 nm in diameter), and apoptotic bodies (ApoBDs) (0.8–5.0 µm in diameter).^18^, ^19^, ^20^ Within the TME, EVs are crucial for intercellular communication, as well as for long‐distance circulation. Although Wolf and colleagues initially regarded EVs as nothing more than cellular waste, emerging data in this area have demonstrated their importance as signaling molecules in physiological and pathological processes, such as cancer development (Figure 1).

Exosome biogenesis is a byproduct of the endosomal pathway. Early endosomes are formed when the cell membrane protrudes inward and their lumens fill with accumulating intraluminal vesicles (ILVs). The development of multivesicular bodies (MVBs) is then initiated by the maturation of early endosomes into late endosomes.^21^ Following their production, MVBs are subsequently transported by cytoskeleton filaments and proteins to the plasma membrane, where SNARE proteins assist in the release of the ILVs as exosomes.^22^, ^23^ The endosomal sorting complex required for transport (ESCRT) has been identified to be involved in MVB biogenesis, vesicle budding, and protein cargo sequestering and sorting.^24^ There is also an ESCRT‐independent pathway that leads to the creation of ILVs and MVBs. Exosomes are commonly referred to as “cargo,” as they encapsulate numerous substances, such as proteins (membrane proteins, cytosolic and nuclear proteins, extracellular matrix proteins), nucleic acids (mRNAs, noncoding RNAs [ncRNAs] and DNA), and metabolites.^21^ There are significant similarities in the types of lipids in EV membranes and the cells from which they originated, such as sphingomyelin, gangliosides, and disaturated lipids.^25^ However, unlike their cell of origin, exosomes have lower concentrations of phosphatidylcholine and diacylglycerol.^26^ In contrast, the proteins in exosomes are more diverse than the lipids, including biogenesis‐related proteins and parental cell‐specific proteins. In the case of tumor‐derived exosomes, they store protumoral proteins and metabolites that promote tumor growth. Similar to protein composition, the nucleic acid composition of exosomes from tumor cells, such as mRNAs, microRNAs (miRNAs), and other small ncRNAs (sncRNA), reflects the characteristics of the parental cell.

MVs are generated through the direct process of budding from the plasma membrane of cells, as opposed to exosomes.^27^ It is now believed that flippases, floppases, scramblases, and calpain change the composition of the phospholipid bilayer.^28^ The cooperation of these enzymes results in the rearrangement of the components of the phospholipid bilayer and the reorganization of the actin cytoskeleton, enabling physical membrane bending as well as more effective MV formation.^29^ The cellular absorption of MVs is hindered by their large size, posing a major obstacle. A recent study found that macropinocytosis, which involves the production of lamellipodia and plasma membrane ruffling, allows cells to ingest huge amounts of fluid outside the cells, hence enhancing the intake of cellular MVs.^30^

Dying cells produce membrane‐bound vesicles known as ApoBDs, formerly considered trash bags but later shown to transfer useful elements. Up to now, researchers have reported phosphatidylserine (PS) as the only marker for identifying ApoBDs.^31^ During the formation of ApoBDs, one of the initial and easily recognizable morphological alterations is the deformation of cells, which manifests as the formation of membrane blebs.^32^ Actomyosin‐mediated contraction and increased hydrostatic pressure within the cell cause the formation of blebs.^33^ Following repeated blebbing and retraction, ApoBDs are released and filled with cellular components and functional molecules, such as DNA, RNA, and proteins.^34^, ^35^ When apoptosis is initiated by therapy, different types of molecules from tumor cell‐derived ApoBDs affect various signaling pathways, which in turn result in various biological effects and promote tumor progression.^36^, ^37^

EVs are membrane‐encapsulated vesicles that contain bioactive molecules ejected by donor cells. Recipient cells absorb EVs through receptor‐ligand interactions, membrane fusion and phagocytosis.^38^, ^39^ The mechanism by which EVs are taken up by the recipient cell is nonrandom in conjunction with transmembrane proteins. Recent research has established that the tetraspanin Tspan8–CD49d complex plays a substantial role in facilitating the attachment of exosomes to recipient cells.^40^ Additionally, intercellular adhesion molecule (ICAM)‐1 is expressed as a receptor molecule on the membrane surface in a proinflammatory milieu, which improves exosome adherence to recipient cells.^41^ Further research is necessary to clarify the mechanisms by which EVs manipulate cancer cell targets and the factors that regulate their fusion.

### 2.2 Molecular cargo of EVs: proteins, nucleic acids (miRNAs, mRNAs, and DNA), lipids, and metabolites

A variety of substances, including proteins, nucleic acids (DNA, mRNA, and ncRNAs), lipids, and metabolites, can be autocrine and/or paracrine, serving as messengers between tumor cells and stromal cells. This section will address the contents of EVs in order to ascertain how EV payloads affect the biological processes in the TME.^42^

#### 2.2.1 Proteins

EVs play a role in a variety of physiological and pathological processes by delivering signaling molecules, such as soluble proteins contained in their lumens, transmembrane proteins, and membrane‐associated proteins.^43^ Tetraspanins (also called four‐transmembrane crosslinked proteins) are a broad family of essential membrane proteins that control the fusion, migration, and adherence of cells, mainly including CD9, CD37, CD63, CD81, CD82 and CD106.^44^ What is more, tetraspanins also include integrins,^45^ ICAM‐1,^46^ major histocompatibility complex (MHC) class II protein^47^ to facilitate the sorting of protein cargoes (tetraspanin‐interacting proteins). The interaction of metalloproteinase CD10 with tetraspanin CD9 can mediate CD10 loading into exosomes, contribute to the redistribution of peptidase from the cell surface to the extracellular microenvironment and effectively regulate the matrix microenvironment in cancer.^44^, ^48^ The integrin α4β1 is mainly present on the surface of erythroid precursors, and it is also absent on the surface of mature red cells. This integrin α4β1 is at least partly cleared from the plasma membranes of reticulocytes by exosomal pathways.^45^ As mature exosomes are enriched in ICAM‐1 and MHC class II proteins, DCs could transfer functional MHC–peptide complexes and ICAM‐1 to other immune cells through secreting exosomes which play an important role in triggering effector T‐cell responses in cancer.^46^, ^47^ Additional critical protein cargoes are membrane transport and fusion‐related proteins, for instance, Ras‐related protein GTPase Rab, and heat shock proteins (HSPs).^43^ GTPases have been found on the inner membranes of exosomes, which are demonstrated to contribute to vesicular traffic and exosome biogenesis by Rab proteins and ectosome formation by Rho/Rac proteins.^47^ HSP family proteins usually act as molecular chaperones and play roles in the balance of proteostasis and proteolysis.^49^ Hsp90 is the major intracellular chaperone in the HSP family which could interact with a variety of intracellular proteins to contribute to the folding and function of corresponding proteins.^49^ Hsp90 is closely associated with poor prognosis in a variety of tumors which are mostly under a condition of hypoxia, acidosis, and nutrient deficiency.^49^ The expression of Hsp90 would increase accordingly in cancer cells which play crucial roles in promoting tumor growth and metastasis in multiple types of cancers, such as breast cancer and pancreatic cancer.^49^ The lack of Hsp90α (a key subtype of Hsp90) in exosomes induces the deficiency of communication from tumor cells to stromal cells which could promote cellular motility.^43^ Besides the above membrane transport and fusion‐related proteins, the surfaces of EVs also contain various other transmembrane proteins with scaffolding functions, for instance, IL‐6,^50^ PD‐L1,^51^ EGFR, T‐cell receptor (TCR),^52^ flotillin 1 and 2,^53^ which are closely related to poor tumor immunity and prognosis. Additionally, the surface of the exosomal membrane harbors many membrane‐interacting proteins, such as proteoglycans and glypican‐1 which are also associated with the tumor progression process.^54^ As members of MVB‐related proteins, ALIX and TSG101 are known as important components of the ESCAT complex and are also the stereotypical biomarkers for exosome characterization.^55^ Furthermore, ALIX and TSG101 proteins could affect the cargo content of EVs.^55^ Based on the signals carried by proteins of EVs, the promise of selective protein cargos as a way to forecast cancer development and evaluate therapy responses has been highlighted by the critical role of proteomic analyses across different cancer types.^56^

#### 2.2.2 RNAs

A large body of literature has shown that EVs enrich certain RNAs, mainly including miRNAs, long ncRNAs (lncRNAs) and circular RNAs (circRNAs).^47^, ^57^ Evidence indicates that RNA‐binding proteins (RBPs) are crucial for the selective sorting of a variety of RNAs. One of the RBPs, for instance, hnRNPA2B1 plays a pivotal role in the regulation of exosomal sorting of tumor cell miRNAs (miR‐198, miR‐601) and lncRNAs (AFAP1–AS1, AGAP2–AS1), which is involved in facilitating colorectal cancer (CRC) and bladder cancer metastasis.^58^, ^59^ Despite the fact that RBPs are crucial for the sorting of RNA into exosomes, the precise mechanisms by which they interact with exosome biogenesis remain obscure.

miRNAs are important members of sncRNAs, which have been extensively studied in the development processes of various cancers.^60^ Numerous exosomal miRNAs can serve as potential biomarkers for cancer prognosis. It was shown that lung cancer patients had extraordinarily abnormally high expression of exosomal miR‐451a and miR‐4257, which is closely associated with cancer development, recurrence, and poor prognosis.^61^ High levels of exosomal miR‐375 and miR‐1290 were also discovered to predict poor prognosis in prostate cancer (PC) patients.^62^ miR‐9 in exosomes is highly associated with breast cancer cell migration by promoting the transformation of normal fibroblasts into cancer‐associated fibroblasts (CAFs).^63^ Furthermore, miRNAs can also serve as potential biomarkers for cancer grading basis and early diagnosis as exosomal miRNAs could be isolated and analyzed from blood or serum. There is evidence showing let‐7a‐5p from plasma EVs was extremely downregulated in high Gleason score (GS) PC patients compared with the patients with low GS.^64^ Exosomal miR‐1246 in serum was considered a potential biomarker for early diagnosis of gastric cancer (GC).^65^ Multiple exosomal miRNAs (miR‐21, miR‐23a, miR‐26, miR‐126) have been identified as noninvasive biomarkers for the diagnosis of cholangiocarcinoma (CCA) or lung cancer.^43^

lncRNAs are broadly classified as RNA transcripts that are >200 nucleotides in length and do not encode protein. lncRNAs could be selectively packaged into EVs and involved in the regulation of tumor growth, migration, metastasis, angiogenesis, and drug resistance.^66^, ^67^ For instance, the high expression level of exosomal lncRNA activated by transforming growth factor β (TGF‐β) in lung cancer, GC, and CRC is strongly associated with tumorigenesis and tumor development by inducing epithelial–mesenchymal transition (EMT).^68^ Jiang et al.^43^ found that exosomal lncRNA H19 secreted by cancer stem cells (CSCs) could then be ingested by endothelial cells and promote tumor angiogenesis by upregulation of VEGF. Additionally, lncRNAs still play a crucial role in drug resistance in cancers. Exosomal Linc00969 increases the production of HER‐2 at the protein level while preserving the stability of HER‐2 mRNA, resulting in trastuzumab resistance.^67^

circRNA is an additional type of ncRNA in EVs, which is generally associated with clinicopathologic characteristics.^69^ Several circRNAs in EVs were found in TME across a range of cancer types, and they act as regulators to mediate interactions between tumor cells and the surrounding tissue as well as various immune cells.^70^ Plasma exosomal circRNA‐002178 has been shown to facilitate the expression of PD‐L1 and induce T‐cell exhaustion in lung cancer patients.^71^ Furthermore, exosomal circRNAs are also involved in mediating tumor angiogenesis, invasion, and metastasis. For instance, the upregulation of circ‐RanGAP1 in plasma exosomes could induce the migration and invasion of GC by promoting miR‐877‐3p/VEGFA axis.^72^ Otherwise, tumor cells could also secrete EVs containing circRNAs, which are strongly associated with drug resistance. Exosomes produced by chemoresistant CRC cells could transport ciRS‐122 and decrease oxaliplatin (OXA) sensitivity in chemosensitive cells.^73^ EVs have also been found to contain mitochondrial RNAs, small nuclear RNA, small nucleolar RNAs, piwi‐interacting (piRNAs), transfer RNA (tRNA) fragments, vault RNAs, Y RNAs, and ribosomal RNA (rRNA) fragments.^57^, ^74^ The transfer of these RNAs allows them to function as regulators or templates for protein synthesis. In addition to the significance of the types of RNAs in EVs, other findings offer a comprehensive understanding of the intricate process of RNA sorting into EVs and underscore the intricate interaction between RBPs, RNA modifications, and the mechanisms involved in EV formation, which has garnered attention in recent years.^75^, ^76^

#### 2.2.3 DNAs

As multiple proteins and RNA were investigated as cargo and delivered through EVs, various DNA were also encapsulated by EVs, including double‐stranded DNA (dsDNA), single‐stranded DNA (ssDNA), and mitochondrial DNA (mtDNA).^77^ According to previous studies, most DNA in EVs was from ApoBDs, and recent studies found some nonapoptotic EVs also emerged as carriers of DNA.^47^ Interestingly, large nonapoptotic EVs seem to contain more DNA in cancers which suggests that EV‐associated DNA might be generated due to the genomic instability of cancer cells.^78^

The presence of DNA within EVs might play a crucial role in liquid biopsy analysis in cancers.^79^ Since Kalluri summarized the discovery of dsDNA in circulating exosomes in 2016,^80^ the following studies examined the exosomal DNA in implications of cancer diagnosis and disease monitoring. For instance, the levels of mutant KRAS DNA were highest in EVs in pancreatic cancer patients with disease progression.^81^ Furthermore, DNA damage induced by antitumor therapy in cancer cells might be associated with exosomal DNA enrichment, and DNA secretion via exosomes might play an important role in cytoprotection since it alleviates the accumulation of deleterious cytoplasmic DNA.^82^ Till now, there has been limited research on the clinical significance of exosomal DNA. Further research is needed to explore the potential clinical implications in cancer diagnosis and therapies of EV‐associated DNA cargo.

#### 2.2.4 Lipids

The lipids including cholesterol, phosphatidylcholine, and glycosphingolipids play important roles in EV biogenesis, uptake and regulating the function of recipient cells.^47^ Recently, researches have shown that lipids are also an important cargo of EVs, besides proteins, DNA, and RNAs. Recent studies have shown there are more than 200 species of EV‐associated lipids derived from cancer cells. For example, targeted molecular lipidomic assays were performed in a metastatic PC cell line (PC‐3) for depth analysis of the lipidomes released by exosomes.^83^ In this study, a remarkable enrichment of various lipids was sorted into EVs, including glycosphingolipids, sphingomyelin, cholesterol, and PS.^83^ Another study reported high‐resolution lipidomic analyses of EVs revealed the difference in the lipid cargo of exosomes and MVs among glioblastoma, hepatocellular carcinoma (HCC) and bone marrow‐derived mesenchymal stem cells (BM‐MSCs).^84^ Furthermore, the differences were found in the lipid cargo of exosomes derived by cancer cells compared with parent cells which suggested the potential clinical implication of early cancer diagnosis.^83^, ^85^ For instance, there was a higher exosomal ceramide expression level isolated from the urine of PC patients compared with healthy patients, which indicated the potential utility of ceramides as fluid‐based biomarkers.^85^ Additionally, the lipids on the surface of EVs are essential for successful drug delivery based on their protection and storage function.^86^

#### 2.2.5 Metabolites

Even though metabolites are constituents of EVs’ cargo, they have received inadequate attention. Since metabolites represent all of the beginnings and ends of biological activities, they may serve as a phenotypic activity of an organism's state.^87^ Thus, tracking metabolic alterations in the patient's bodily fluids—such as blood, urine, synovial fluid, saliva, and cerebrospinal fluid—may offer insightful diagnostic evidence regarding the state of the illness and the effectiveness of treatment. In a pilot study, Wojakowska et al.^88^ compared the metabolite patterns of serum exosomes between healthy controls and patients who received radiation therapy to reveal the effects of radiation on patients with head and neck cancer. They found that exosome samples from patients and health control differed in the ratio of metabolites related to energy production, such as the Warburg effect, glycolysis, pyruvate metabolism, and the mitochondrial electron transport chain. Numerous studies have demonstrated that recipient cells undergo metabolic alterations as a result of EVs produced from tumors and tumor stroma.^89^, ^90^ However, the biological significance of EV metabolites in malignancies is currently poorly understood compared with proteins and nucleic acids. The two issues that may be causing this are the absence of an effective EV extraction methodology to produce high‐purity EVs that eliminate nonexosomal metabolites found in the biofluidic matrix, as well as the shortcomings of existing methods to precisely evaluate and validate EV metabolomes.^91^

## 3 MECHANISMS OF EV‐MEDIATED INTERACTIONS IN CANCER

### 3.1 EV‐mediated transfer of oncogenic signals between cancer cells

**FIGURE 2:**
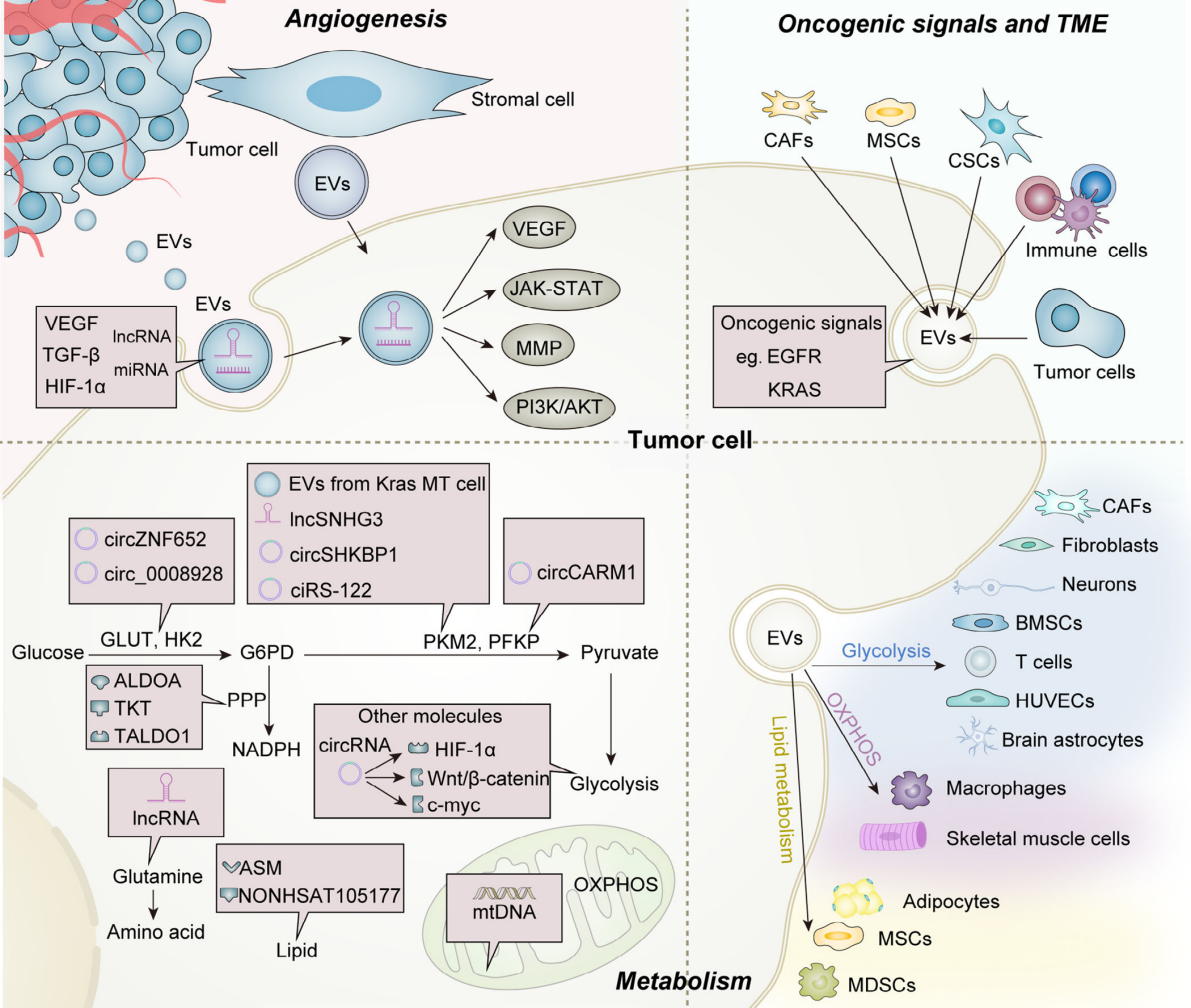
Functions of TDEVs in tumor progression. The figure highlights the current understanding of the roles of EVs during different stages of cancer including angiogenesis, oncogenic signaling, TME remodeling, and metabolic reprogramming. EVs carrying diverse factors in the TME enhance critical signaling pathways in tumor cells related to angiogenesis. Cargos (such as EGFR and KRAS) derived from TME (mainly from CAFs, immune cells, MSCs, and tumor cells) to other tumor cells, thereby stimulating the oncogenic signaling pathway in recipient cells. Cancer cells exhibit a significant dependence on glucose as their predominant energy substrate, with the regulation of glycolysis being governed by rate‐limiting enzymes, including PKM2, PFKP, and HK2. EVs regulate glycolysis through these key enzymes and other essential molecules targeted by EVs. The deregulation of amino acid, lipid metabolism, and increased OXPHOS via EVs‐mediated processes is being more recognized as a characteristic of cancer cells, presumably playing a role in the malignant development. TDEVs are thought to have a role in the metabolic reprogramming of neighboring cells, including CAFs, MDSCs, T cells, macrophages, and other various cell types such as fibroblasts, brain astrocytes, skeletal muscle cells, HUVECs, MSCs, and adipocytes. EVs, extracellular vesicles; TME, tumor microenvironment; EGFR, epidermal growth factor receptor; KRAS, Kirsten rat sarcoma virus; OXPHOS, oxidative phosphorylation; TDEVs, tumor‐derived EVs; CAFs, cancer‐associated fibroblasts; MDSCs, myeloid‐derived suppressor cells; HUVECs, human umbilical vein endothelial cells; MSCs, mesenchymal stem cells.

Cancer cells display high heterogeneity in terms of molecular signatures within the same tumor site. Therefore, EVs released from cancer cells can function as a novel kind of messenger by transferring essential oncogenic signals to other cancer cells, which in turn promotes tumor growth (Figure 2). EGFR is an important oncogenic component of cell signaling pathways that regulate cell division and survival.^92^ Mutations in the EGFR gene result in the higher expression of EGFR proteins in certain types of cancer cells and transportation of EGFR via EVs induces an accelerated progression of cancer cells. For instance, glioma cells transfer oncogenic EGFR variant III (EGFRvIII) via EVs to other glioma cells lacking this receptor, promoting their morphological transformation and cell proliferation by activating the MAPK and AKT signaling pathway.^93^ Another in vitro study also reported that EGFRvIII derived from EVs can stimulate the proliferation of human U87 glioma cells.^94^ GC can spread to the liver more easily since exosomes carrying EGFR have an impact on the liver microenvironment.^95^ In addition to promoting the proliferation of recipient cells, EVs also play pivotal roles in cancer chemoresistance and radiotherapy resistance by horizontal transfer of resistance‐related molecules to nonresistant cancer cells. In a non‐small cell lung cancer (NSCLC) model, Wu et al.^96^ suggest that EV‐mediated transfer of wild‐type EGFR protein promotes osimertinib resistance to EGFR‐mutated sensitive cancer cells by activating PI3K/AKT and MAPK signaling pathways.

EVs also contain other molecules that are critical for the oncogenic signaling pathway. A highly malignant pancreatic cell line PC‐1.0 could enhance the proliferation of another moderately malignant cell line PC‐1 through transferring oncogenic ZIP4 via EVs.^97^ It has also been reported that EVs from KRAS mutant colon cancer cells transfer mutant KRAS to wild‐type cells resulting in their enhanced three‐dimensional growth.^98^ In another lung cancer model, highly metastatic lung cancer cells 95D strongly accelerated the proliferation and migration of poorly metastatic lung cancer cells 95C, which was mediated by the EV‐transferred hepatocyte growth factor (HGF).^99^ Taken together, these studies demonstrated that tumor‐derived EVs (TDEVs) can contribute to the horizontal propagation of oncogenic signals among subsets of cancer cells, which induces a more aggressive phenotype of the recipient cancer cells and results in tumor progression.

### 3.2 EV‐mediated communication between cancer cells and the TME

Cancer cells also interact with noncancer cells in the TME by EV‐mediated signaling, particularly with CAFs, a prevalent kind of stromal cells. The dynamic interaction between cancer cells and CAFs plays a crucial role in tumor formation and partially relies on the transmission of signals through EVs. On one hand, TDEVs are capable of transforming or activating CAFs. Webber et al.^100^ reported that some cancer cells secrete EVs containing TGF‐β which could transform normal stromal fibroblasts into CAFs. Multiple other studies also demonstrated that functional cargoes such as TGF‐β, miR‐1247‐3p, and miR‐125b transferred by EVs from liver, bladder, and breast cancer cells, respectively, induced the activation of CAFs.^101^, ^102^, ^103^ As a result, the activated CAFs promote cancer cell proliferation and invasiveness by remodeling the extracellular matrix within the TME or releasing soluble factors.^104^, ^105^ On the other hand, EVs secreted by CAFs can also mediate tumor aggressiveness. EVs derived from CAFs isolated from human oral squamous cell carcinoma (OSCC) significantly induce migration and invasion of OSCC cells in vitro.^106^ Similarly, breast‐cancer‐associated fibroblasts release EVs that activate Wnt‐planar cell polarity signaling, enhancing the protrusive activity and motility of breast cancer cells.^107^ To explore the role of EVs from CAFs involved in chemotherapy resistance, Bai et al.^108^ demonstrated that EVs enriched with miR‐522 are transmitted from CAFs to chemo‐sensitive cancer cells in GC. Upon assimilation, miR‐522 reduces the expression of arachidonate lipoxygenase 15 (ALOX15), thus decreasing the lipid peroxides accumulation and resulting in chemo‐resistance of recipient cancer cells.^108^ In addition to transferring functional biomolecules such as proteins and miRNAs, CAF‐derived EVs can directly supply nutrients for starved cancer cells. It has been shown that EVs secreted by patient‐derived CAFs contain intact metabolites including amino acids, lipids and TCA‐cycle intermediates. These substances can be used by cancer cells to support their central carbon metabolism when they are lacking nutrients, ultimately promoting tumor growth.^109^ In the TME, MSCs are highly regarded as a promising type of stem cells in the field of tissue engineering due to their convenient accessibility and their capacity to differentiate into many cell types such as adipocytes, osteoblasts, cardiomyocytes, and neurons.^110^ MSC‐derived exosomes in the TME bestow colorectal stem cell characteristics by activating ERK1/2 and stimulating the Wnt signaling pathway, which raise the proportion of CSCs and promote the formation of tumor spheroid in vitro and tumorigenicity in vivo.^111^ BM‐MSCs released exosomes that contained miR‐214 inhibited oxidative stress injury in CSCs, which ultimately assisted in the formation of tumors by means of CaMKII silencing.^112^ Exosomes derived from tumor‐associated MSCs exhibited elevated levels of miR‐155. This, upon absorption by tumor cells, could lead to the inhibition of tumor suppressor genes SMARCA4 and augment the potential of tumor migration.^111^ Furthermore, it has been discovered that glioma‐associated human MSCs, which are a possible novel target in glioblastoma, can enhance the invasiveness of glioma stem cells through exosome‐derived miR‐1587.^113^ Nevertheless, it is intriguing that it presents antiangiogenic miRNAs in MSC exosomes, such as miR‐16 and miR‐100, which block angiogenesis in breast cancer cells by targeting VEGF.^113^, ^114^

CSCs are a subset of heterogeneous cells residing in tumor sites possessing an unlimited capacity for self‐renewal and diversification.^115^ EVs have a role in transferring information to facilitate the conversion between non‐CSCs and CSCs.^116^ They could participate in maintaining the balance of CSCs such as lncRNA FMR1‐AS1 in exosomes through TLR7‐NFκB signaling activation.^117^ A number of processes, such as improved DNA repair efficiency and antiapoptotic ability, slowed cell cycle progression, drug efflux, and production of detoxifying enzymes, contribute to CSC therapeutic resistance, where EVs play a significant role in this process.^118^ As drug efflux pumps contribute to multidrug resistance (MDR) of cancer cells, the transfer of those transporters from drug‐resistant cancer cells to low‐expressing drug‐sensitive cancer cells via EVs serves as an important scheme of resistance propagation. For instance, one of the drug efflux pumps P‐glycoprotein plays a key role in maintaining intracellular drug concentration. Several studies found that EVs isolated from chemoresistant osteosarcoma, breast, ovarian, and PC cells transport P‐glycoprotein to drug‐sensitive cells and mediate the extrusion of drugs to enhance their chemoresistance.^119^, ^120^, ^121^, ^122^ Stromal cells are responsible for orchestrating a complex interaction with breast cancer cells in order to regulate the growth of therapy‐resistant tumor‐initiating cells. This is accomplished through the transfer of exosomes, which increases the expression of the IRDS genes (Interferon‐Related DNA Damage Resistance Signature).^123^ In summary, all these studies support the role of EVs in the crosstalk between cancer cells and stromal cells within the TME. Furthermore, the function of EVs between cancer cells and immune cells within the TME is discussed further below.

### 3.3 EV‐mediated metabolic reprogramming in tumors

#### 3.3.1 EVs facilitating the metabolic reprogramming between tumor cells

Cancer cells exhibit a preference for glycolysis as their primary energy production pathway, even when oxygen is readily available. Therefore, aerobic glycolysis has been considered a characteristic that separates cancer cells from noncancer cells. The M2 isoform of pyruvate kinase 2 (PKM2), a major limiting glycolytic enzyme, provides substantial nutrients for cancer metabolism and progression.^124^, ^125^, ^126^ The process by which exosomes carry enzymes, metabolites, and ncRNAs plays a key role in PKM2 expression and metabolic shifts in cancer. A recent study demonstrated that exosomes containing lncRNA SNHG3 released by CAFs blocked mitochondrial oxidative phosphorylation (OXPHOS) and boosted glycolysis; exosomes were taken up by breast cancer cells, and the delivered SNHG3 increased PKM expression by sponging miR‐330‐5p.^127^ The pentose phosphate pathway (PPP) is an additional branch of glycolysis that is responsible for rerouting glucose to create ribose‐5‐phosphate and nicotinamide adenine dinucleotide phosphate. Proteomic analysis revealed that G6PD, TKT, and TALDO1, three dominant enzymes of the PPP, were enriched in ovarian cancer‐derived exosomes and could serve as diagnostic and therapeutic biomarkers.^128^ Numerous studies have investigated the role of PKM2 in EVs; however, its therapeutic use in clinics is limited. In addition to the crucial enzymes involved in glycolysis, EVs have the potential to modulate glucose metabolism via nucleic acids (such as miRNAs, lncRNAs, and circRNAs), which affect protein expression in specific cells (Figure 2).

Recent research has shown that cancer cells rely on glutaminolysis to meet their biochemical requirements, while immune cells preferentially absorb glucose (in comparison with cancer cells).^129^, ^130^, ^131^ This finding highlights the possibility that cancer cells conditionally prefer certain “addicting” nutrients in the TME. Glutaminase metabolism, mediated by glutaminase 1 (GLS1), contributes to the tumor‐promoting phenotypes induced by circTRPS1 in bladder cancer. By way of the circTRPS1 inducing GLS1 via binding to miR141‐3p, exosome‐derived circTRPS1 was capable of regulating the intracellular ROS equilibrium.^132^ Currently, the dysregulation of lipid metabolism through exosome‐mediated mechanisms is being increasingly acknowledged as a hallmark of cancer cells, potentially contributing to the malignant progression and metastatic tendencies.^133^ In pancreatic ductal adenocarcinoma (PDAC) cells, exosomes containing NONHSAT105177 reduced the factors involved in cholesterol biosynthesis.^134^ In the TME, a large quantity of exosomes is secreted by adipocytes and carry proteins that are involved in fatty acid oxidation (FAO). Studies have demonstrated that melanoma cells take up adipocyte exosomes, resulting in an increase in FAO and FAO‐dependent migration and invasion.^135^ Lipidomic analysis of the plasma of patients with MM revealed dysregulation of sphingolipids metabolism, characterized by the upregulation of multiple ceramides and the downregulation of sphingomyelin.^136^ MM exosomes were shown to encapsulate the enzyme acid sphingomyelinase (ASM), which was responsible for sphingolipids metabolism and confers drug resistance to recipient cells.

Based on the finding that cancer cells exhibit elevated glycolysis in comparison with healthy cells, OXPHOS is thought to be generally downregulated in cancer. In contrast to previous assumptions, recent findings have demonstrated that numerous tumors exhibit a significant reliance on OXPHOS for the synthesis of ATP. Among breast cancer subtypes, luminal breast cancer cells prefer effective mitochondrial respiration to maintain their capacity to cause tumors, while triple‐negative and HER2+ breast cancer cell lines have a greater reliance on anaerobic glycolysis. OXPHOS is essential for the reactivation of ER+ breast cancer cells from dormancy, as is the presence of mitochondrial complex proteins and mtDNA. Bromberg et al.^137^ reported that CAF‐derived EVs containing whole genomic mtDNA mediated the restoration of OXPHOS and the acquisition of hormonal therapy resistance in ER+ cells. Cancer cells can maintain a precise equilibrium between aerobic respiration and glycolysis to evade the detrimental consequences caused by the overproduction of ROS resulting from OXPHOS.^138^

Metabolic reprogramming in cancer has been substantially elucidated beyond the “Warburg effect” as a result of the comprehensive investigation of tumor metabolism, emphasizing the importance of lipid, amino acid, and OXPHOS metabolism.

#### 3.3.2 Reshaping the metabolism of different cells in the TME

Recent reports have shed light on the involvement of EVs produced by tumor cells in the metabolic reprogramming of nonneoplastic cells (Figure 2). CAFs are significant biological components of the tumor stroma. EVs facilitate communication among CAFs, tumor cells, and other stromal cells. TDEVs are thought to play a role in metabolic reprogramming by transferring EV‐encapsulated bioactive molecules, which posttranscriptionally control gene expression in CAFs. When there is an adequate supply of nutrients in the TME, CAFs are thought to increase glucose and glutamine metabolism via EV‐containing miR‐105. Under nutrient‐limited conditions, miR‐105 primed CAFs transform metabolic wastes such as lactic acid and ammonium into high‐nutrient compounds.^139^ Factors from tumors may cause local quiescent fibroblasts to differentiate into various subgroups of functional CAFs, each of which has a unique protein expression pattern and secretory profile. Compared with normal fibroblasts which utilize OXPHOS, CAFs utilize aerobic glycolysis that is triggered in response to signals from tumor cells, which is known as the “reverse Warburg effect.” Neonatal human foreskin fibroblasts (neoHFFs), a type of normal fibroblast stimulated by EVs, have been shown to overexpress GLUT1 and MCT4, which is especially important for enhancing glucose and lactate uptake by CAFs in the TME.^140^ These findings indicate that the exchange of EVs between cancer cells and CAFs significantly influences metabolic processes within cells, highlighting the importance of EV‐mediated metabolic reprogramming in intercellular communication.

Tumor cells could be shielded from chemotherapy by vascular endothelial cells. Research showed that acute myeloid leukemia (AML) cells produced exosomes containing VEGF and VEGFR, which stimulate the basal and compensatory glycolysis in HUVECs.^141^, ^142^ These discoveries might help to pave the way for the creation of innovative therapeutic techniques that target exosomes in AML.^143^ Due to the unique properties of MSCs, they are extraordinarily promising for the field of cell‐based cancer therapy. Researchers have employed metabolomics to investigate the metabolites of EVs derived from human MSCs. Apart from adipocytes themselves, human adipose tissue‐derived MSCs (hAD‐MSCs) are also involved in lipid metabolism. The TGF‐β signaling pathway has been implicated in the inhibition of hAD‐MSC adipogenesis induced by lung tumor‐derived exosomes.^144^ As the primary biological component of the TME, adipocytes undergo transformation into cancer‐associated adipocytes, which subsequently promote tumor progression.^145^, ^146^ The potential ability of adipocytes to promote the growth of tumors may be attributed to their metabolic reprogramming as a result of the dynamic interaction between cancer cells and adipocytes through EVs. Adipose tissue lipolysis contributes to early weight loss. Exosomes released by pancreatic cancer cells cause lipolysis in adipocytes through the delivery of adrenomedullin, a potential modulator of adipose tissue that binds to its receptor, triggering p38 and ERK1/2 MAPKs and phosphorylating hormone‐sensitive lipase.^147^ Cancer‐associated cachexia (CAC) is characterized by a loss of adipose tissue, which occurs mostly as a result of increased lipolysis and impaired adipogenesis. Exosomes derived from Lewis lung carcinoma (LLC) cells also induce adipocyte lipolysis both in vitro and in vivo, which implies that inhibiting exosomes could serve as a potential CAC treatment method.^148^

TDEVs have the potential to engage with a variety of immune cell metabolisms in TME. Golab et al.^149^ identified a unique strategy of T cell malfunction based on the activity of ovarian cancer cells‐derived EVs transferring arginase 1 (ARG1). RNA‐seq analysis, performed after exosome delivery, highlighted the dynamic changes that had occurred in the transcriptome of CTLL2 cytotoxic T cells. When transiently coexpressed gene clusters were analyzed, pathway enrichment revealed that the B16F0 exosomal payload affected T‐cell mitochondrial respiration.^150^ PC exosomal IL‐8 stimulated the excessive activation of peroxisome proliferator activated receptor alpha (PPARα) in CD8+ T cells, leading to a decrease in glucose utilization by reducing the expression of GLUT1 and HK2. Moreover, it also resulted in an increase in fatty acid breakdown by boosting the expression of CPT1A and ACOX1. Instead of adenosine triphosphate (ATP) production, PPARα further activates uncoupling protein 1 (UCP1), which induces fatty acid catabolism for thermogenesis. Thus, by neutralizing the impact of exosomal IL‐8, the suppression of PPARα and UCP1 promoted CD8+ T‐cell proliferation.^151^ Metabolic alteration plays a critical role in the exhaustion of CD8+ T cells induced by cancer cells. Myeloid‐derived suppressor cells (MDSCs) in the TME aid in the tumor progression by disturbing cytotoxic T‐cell response and mediating immune evasion, partially relying on tumor‐associated factors such as prostaglandin E2 (PGE2). PGE2 is a derivative of arachidonic acid, acting as a highly effective lipid mediator to regulate lipid metabolism. A study reported that tumor‐secreted exosomes were more PGE2 loaded, which ultimately led to the accumulation of MDSCs and the promotion of tumors.^152^ Besides that, exosomes reprogram the metabolism of tissue‐resident macrophages, which causes them to adopt an immunosuppressive phenotype in a premetastatic environment. As a result of exosome signaling, NF‐κB serves as the primary transcription factor that employs HIF‐1α and subsequently GLUT‐1 to facilitate increased glucose uptake in macrophages while also utilizing NOS2/NO to impede mitochondrial OXPHOS.^153^ When treated THP‐1 with celecoxib‐treated lung cancer cell culture supernatant, the absorption of COX‐2 by monocytes via exosomes increased the synthesis of PGE2 and VEGF.^154^ In numerous studies these days, exosomes produced from tumors may do the explanation by altering distant sites’ metabolism and encouraging metastases at these locations. A typical metastatic target of advanced PC is bone metastases.^155^ Uncertain mechanisms underlie PC's propensity to produce clinically apparent bone metastases. Through the exosome‐mediated transfer of PKM2 into BM stromal cells by primary PC cells and the consequent upregulation of CXCL12, primary PC cells instruct the BM to develop a premetastatic niche.^156^ As a result, a feedback loop is established by cancer cells, which regulates the metabolism of stromal cells, thereby promoting the proliferation of cancer cells.

### 3.4 Role of EVs in promoting angiogenesis

Angiogenesis is a complex and dynamic process by which tumors develop new blood vessels to supply oxygen and nutrients, thus playing a critical role in tumor growth and progression. The VEGF which can induce vascular permeability and tube formation is the most typical regulator that initiates angiogenesis.^157^ TDEVs can induce angiogenesis by horizontal transfer of VEGF directly or molecules regulating the VEGF pathway. For example, VEGF‐A exhibits a higher capacity of permeability and angiogenic potential of human brain endothelial cells carried by glioblastoma stem‐like cell‐derived EVs.^158^ A VEGF isoform localized on the surface of EVs can also promote tumor angiogenesis by stimulating endothelial cell migration and tube formation.^159^ Another glioma cell U87‐MG‐derived EVs transfer linc‐CCAT2 to endothelial cells, promoting human umbilical vein endothelial cells (HUVECs) migration, proliferation and tubular‐like structure formation by upregulating VEGFA expression.^160^ Similarly, EVs containing miR‐25‐3p can be transferred from CRC cells to endothelial cells, promoting vascular leakiness and enhancing CRC metastasis by regulating the expression of VEGFR2 which is the main signaling receptor for VEGF.^161^ There are also other factors transferred by EVs that can promote angiogenesis without stimulating the VEGF signaling pathway. For example, treated with anti‐miR‐9 or JAK inhibitor, EV‐induced angiogenesis was suppressed by increasing the SOCS5 levels and deactivating the JAK–STAT signaling pathway.^162^ CCA‐derived EVs transfer circ‐CCAC1 from cancer cells to endothelial monolayer cells, disrupting endothelial barrier junction and promoting angiogenesis by increasing GRB2‐like protein 2 expression.^163^ Angiogenesis is also greatly impacted by TGF‐β, especially TGF‐β‐enriched exosomes.^164^ TGF‐β+ EVs from HNSCC facilitate the transformation of nonactivated macrophages into the proangiogenic M2 phenotype.^165^ Blocking TGF‐β interactions could make these TGF‐β‐enriched exosomes attractive targets for antiangiogenic treatment. Similar to bevacizumab, a popular angiogenesis inhibitor that targets VEGF, RER is a recently created TGF‐β inhibitor that binds to TGF‐β and greatly reduces angiogenesis.^166^ Apart from cancer cell‐derived EVs, other cell types within the TME can also shed EVs to mediate angiogenesis. For example, miR‐10a‐5p from CAF‐derived EVs promotes angiogenesis in vivo and in vitro by activating the Hedgehog signaling in cervical squamous cell carcinoma.^167^ The delivery of miR‐21 to multiple myeloma (MM) endothelial cells by CAF‐derived EVs was demonstrated in another study, which subsequently facilitated angiogenesis.^168^

There are substantial variations in the protein content of TDEVs in different types of cancer, including different proangiogenic factors. According to molecular characterization, EVs generated from glioblastoma have all the necessary components for stimulating angiogenesis including angiogenin, VEGF, TGF‐β, IL‐6, and IL‐8.^169^, ^170^ Additionally, exosomes are exceptionally abundant in CD44 variant isoform 5, ICAM‐1, and MMP‐13 in nasopharyngeal carcinoma. Conversely, these exosomes exhibit downregulation of the angiosuppressive protein thrombospondin‐1 (TSP‐1).^171^, ^172^ By serving as a coreceptor for tissue plasminogen activator, exosomal annexin II stimulates angiogenesis in breast cancer while exosomes from bladder cancer exhibit overexpression of EGF‐like repeats and discoidin I‐like domain‐3, which are critical for angiogenesis.^173^, ^174^ Moreover, the following proteins are found in exosomes produced by MM as proangiogenic factors: VEGF, basic fibroblast growth factor, MMP‐9, HGF, and serpin E1.^175^ Proangiogenic proteins such as endothelin‐1, IL‐8, and VEGF could be induced in lung cancer by exosome‐derived sortilin.^176^

Hypoxia is a crucial factor that affects the formation, release, and composition of EVs, as well as cancer angiogenesis. Synergistically, HIF‐1 enhances the expression of proangiogenic factors, including VEGF, and angiopoietin 1/2, placental growth factor.^177^ HIF‐dependent VEGF stimulation and subsequently angiogenesis result from the loss of tumor suppressor genes such as p53, p21, pRb, or PTEN.^178^ Under hypoxic conditions, certain important proteins such as HIF‐1α, lysyl oxidases, plasminogen activator inhibitor 1, platelet‐derived growth factors, TSP‐1, caveolin‐1, annexin II, and signal transducer and activator of transcription 3 are highly transported to exosomes.^179^, ^180^, ^181^, ^182^, ^183^, ^184^ These proteins play a crucial role in modifying the TME, facilitating the progression of the tumor, evading the immune system, promoting angiogenesis and developing resistance to therapy.

Collectively, all these studies suggest that EVs from distinct cells including tumor cells and stromal cells participate in the angiogenesis process by transferring functional cargoes which stimulate VEGF‐dependent or independent signaling pathways, finally leading to tumor progression.

### 3.5 The functions of EVs in mediating immune evasion

**FIGURE 3:**
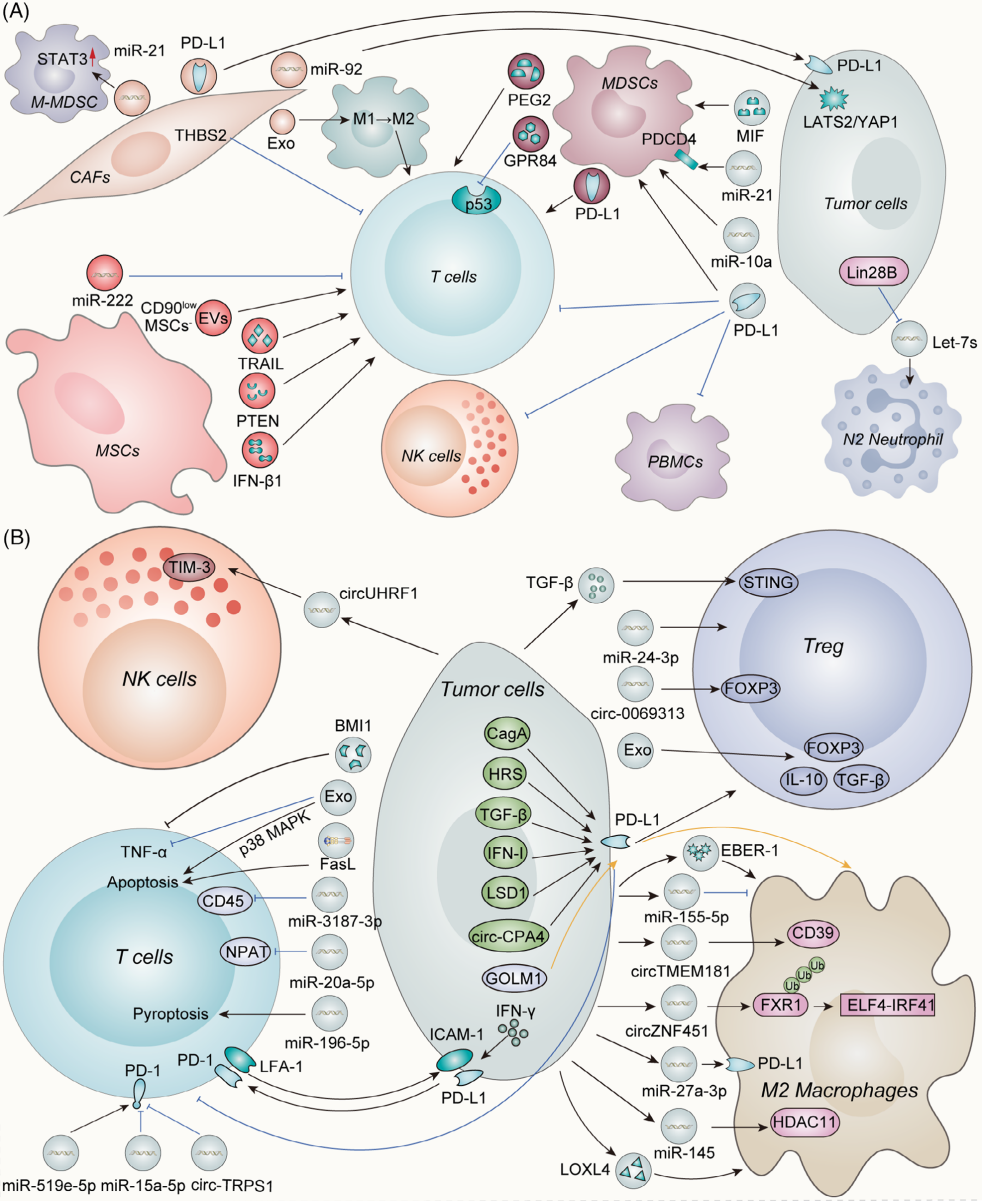
The immunomodulatory role of EVs in the TME. The regulation of the immune microenvironment is significantly influenced by EVs. (A) EVs have a crucial role as bioactive molecules in the interaction between tumor cells and T cells, primarily promoting the amplification of Treg cells and hindering the function of CTL cells. Polarization of M2 macrophages and inactive NK cells are indicators of an immunosuppressive microenvironment generated by the cargo of EVs. (B) There are a number of mechanisms via which EVs influence other stromal cells such as CAFs, MDSCs, MSCs, and neutrophils. As a result, EVs empower their ability to stimulate tumors. However, there is the possibility that MSC‐EVs could be utilized in immunotherapy to stimulate CD8+ T cells. EVs, extracellular vesicles; Tregs, the regulatory T cells; CTL, cytotoxic T lymphocyte; NK, natural killer; CAFs, cancer‐associated fibroblasts; MDSCs, myeloid‐derived suppressor cells; MSCs, mesenchymal stem cells.

EVs that originate from malignant cells are vital targets within the complex web of tumor immunity.^185^ Supporting tumor cells against regulation by immune cells, enhancing tumor cell immune tolerance, and allowing for the evasion of immune surveillance are all attributes of these EVs, which can also inhibit immune function and promote the differentiation of regulatory T cells (Tregs), MDSCs, and tumor‐associated macrophages (TAMs).^186^, ^187^ The purpose of this section is to shed light on the most important aspects of the current landscape of EVs that redefine the immune microenvironment (Figure 3).

#### 3.5.1 T cells

TDEVs have the potential to engage with a variety of immune cells in the TME. Activated CTLs can eradicate tumor cells, where exists a positive correlation between their abundance and an enhanced clinical prognosis. The function of CTLs can be perturbed by a number of processes that result in an exhausted phenotype, which is incapable of reacting to the presentation of antigens. Tumor cell‐surface expressed PD‐L1 and CD8+ T cell‐surface expressed PD‐1 interact through their extracellular structural domains to ultimately impair T‐cell activity.^188^ Mechanistically, PD‐L1 was able to be transported by exosomes^51^, ^189^, ^190^, ^191^, ^192^ or microparticles^193^ secreted by tumors to deactivate T‐cell activities, possibly leading to immunotherapy resistance. Research has shown that a precondition for exosomal PD‐L1‐mediated immune suppression is the interaction between ICAM‐1 on TDEVs and LFA‐1 on activated T cells.^194^ Apart from TDEV‐derived PD‐L1, TAM‐derived exosomes also contain large amounts of PD‐L1 and effectively inhibit the activity of CD8+ T cells.^195^ By stimulating the accumulation of PD‐L1 in exosomes, histone lysine‐specific demethylase 1 (LSD1) decreases the percentage of CD8+ T cells in the microenvironment of GC cells, making LSD1 a novel target for immunotherapy against GC.^196^ CD8+ T cells have not been found to present in areas of melanoma tissue samples where the tumor cells have high levels of phosphorylated HGF‐regulated tyrosine kinase substrate (HRS), an essential element of the ESCRT complex that is implicated in the biogenesis of exosomes. HRS has been shown to interact with PD‐L1 to facilitate its loading onto exosomes, hence impeding the migration of CD8+ T lymphocytes into tumors.^197^ Elevated levels of exosomal PD‐L1 may suggest a negative prognosis for GC patients infected with *Helicobacter pylori*. Functionally, cytotoxin‐associated gene A enhances the expression of PD‐L1 in exosomes obtained from GC cells by impeding the activity of p53 and miR‐34a, hence diminishing the proliferation and anticancer efficacy of CD8+ T cells.^198^ Several pathways, such as the interferon (IFN)‐I pathway^199^ or TGF‐β signaling pathway,^200^ could act upstream of cancer‐derived exosomal checkpoint receptor ligands to trigger their secretion or induce the expression in the exosome, leading to a high tumor burden.

ncRNAs in TDEVs have been demonstrated to be able to downregulate T‐cell responses through decreased TCR signaling and decreased cytokine, granzyme B and perforin secretion.^201^, ^202^ miR‐3187‐3p was shown to inhibit CD45 expression when transported by melanoma exosomes, which is critical to the efficacy of antigen receptor signaling in T cells.^203^ miR‐181 and miR‐498 from TDEVs specifically bind to the 3′UTR of tumor necrosis factor‐alpha (TNF‐α) to directly decrease its expression, thereby reducing its production by CD8+ T cells.^203^ The exosomes generated from papillary thyroid carcinoma include miR‐519e‐5p, which can significantly reduce the expression of granzyme B and increase the expression of PD‐1, leading to the apoptosis of CD8+ T‐cells.^204^ The expression of miR‐196‐5p was significantly elevated in exosomes released by NSCLC cells, which promoted pyroptosis in T cells and exacerbated tumor progression.^205^ CD8+ T cells were rendered dysfunctional as a consequence of the internalization of exosomal miR‐20a‐5p in triple‐negative breast cancer (TNBC), which decreased the expression of the nuclear protein ataxiatelangiectasia in CD8+ T cells.^206^ miR‐24‐3p‐enriched exosomes promoted the formation of the Tregs while increasing T‐cell apoptosis in AML.^207^ Exosomes released by tumor cells have the potential to alter gene expression and immune regulation in host‐receiving cells by delivering circRNAs. Repression of the malignant phenotype of bladder cancer cells was achieved by exosomes from circTRPS1 knockdown bladder cancer cells, which also prevented CD8+ T cells from becoming exhausted.^132^ Additionally, circ‐CPA4 inhibited CD8+ T cells in a PD‐L1‐dependent manner.^208^ O‐GlcNAc transferase, a nutrient sensor that regulates glycolysis and lipid metabolism in cancer, could be contained in exosomes derived from aldehyde dehydrogenase (ALDH) positive esophageal carcinoma stem cells and was capable of penetrating adjacent CD8+ T cells and upregulating PD‐1 expression in CD8+ T cells.^209^ Both growth‐promoting and growth‐inhibiting miRNAs can be found in exosomes secreted by tumor cells. For instance, exosomal miR‐15a‐5p derived from HCC cells halted the progression of HCC by inhibiting PD‐1 expression in CD8+ T cells.^210^ These results emphasize the potential of EV‐derived ncRNAs as promising therapeutic targets to improve the effectiveness of immunotherapies. What is more, a novel approach can be devised by concentrating on these growth‐inhibiting ncRNAs.

EVs from cancer patients can inhibit T‐cell activation and induce apoptosis.^211^ Research has demonstrated that EVs produced by pancreatic cancer cells trigger the p38 MAPK signaling cascade, leading to the apoptosis of CD8+ T lymphocytes.^212^ Through a potential interaction with Fas/FasL, exosomal FasL derived from human PC cells has the potential to induce apoptosis in CD8+ T‐cells.^213^ Tumor antigens could be transferred to lymph node lymphatic endothelial cells by EVs, resulting in the apoptosis of tumor antigen‐specific CD8+ T cells.^214^ As a result, EVs are capable of inducing T‐cell apoptosis in addition to inhibiting T‐cell function.

Tregs, a specialized subpopulation of T cells, maintain homeostasis and self‐tolerance to self‐antigens and especially inhibit the immune response in cancer.^215^ Exosomes extracted from patients with acute lymphoblastic leukemia (ALL) induced apoptosis in T lymphocytes and modulated the T‐cell profile to become Treg through the upregulation of FOXP3, IL‐10 and TGF‐β. Following treatment, interleukins (IL‐17 and IL‐23) and Th17‐related transcription factors (RoRγt) also exhibited decreased expression levels, demonstrating the presence of immunosuppressive molecules in exosomes derived from the serum of ALL patients.^216^ EVs produced by different breast cancer subtypes have varying immunomodulatory properties. Circulating EVs from patients with TNBC exhibit the most immunosuppressive phenotype with a decrease in CD3+HLA‐DR+ T cells and an increase in CD4+CD127‐CD25^hi^ Treg cells.^217^ Unfavorable outcomes among patients with cervical cancer were independently associated with elevated levels of intratumoral STING expression and Treg infiltration. Treg expansion resulted from exosomal TGF‐β‐induced STING signaling in tumor‐infiltrated T cells.^218^ TDEVs transport PD‐L1, which stimulates the differentiation of M2 macrophages and the activation of Treg.^219^ In addition, exosomal circ‐0069313 is capable of impeding the degradation of FOXP3 and thereby enhancing the functionality of Treg when transferred to these cells.^220^ However, the mechanisms underlying tumor‐associated Treg expansion are heterogeneous, indicating that further investigation is warranted.

#### 3.5.2 MDSCs

Regarding the interaction and underlying mechanisms between MDSCs and cytotoxic T cells within the TME, EVs play a significant role in the dysfunction of cytotoxic T cells induced by MDSCs.^221^, ^222^ Exosomes derived from MDSCs (MDSC‐Exos) caused excessive activation or depletion of CD8+ T cells, leading to increased formation of reactive oxygen species (ROS) and ultimately resulting in activation‐induced cell death in CD8+ T cells.^223^ PD‐L1 is also abundant in MDSC‐Exos reducing the antitumor activity of CD8+ T cells.^224^ T‐cell senescence is induced via the p53 signaling pathway when GPR84 is transferred from MDSCs to CD8+ T cells; this may account for the potent immunosuppressive effects of GPR84 in MDSCs.^225^ According to a substantial body of evidence, TDEVs are implicated in the immunosuppressive activity of MDSCs within the TME. However, the underlying mechanisms remain elusive. Recent research reported that macrophage migration inhibitory factor (MIF) played a critical role in the differentiation of MDSCs caused by exosomes in pancreatic cancer. As a result, MIF tautomerase inhibitors have the ability to counteract the immunosuppressive milieu of pancreatic cancer and boost anticancer immune responses.^226^ Melanoma‐derived EVs triggered an immunosuppressive response in the immune system. This response was characterized by a decrease in NK cells and CD8+ T cells in the spleen and BM, an increase in Treg in lymph nodes, and an increase in polymorphonuclear MDSCs in the BM.^227^ Furthermore, downregulation of the protein programmed cell death protein 4 by exosomal miR‐21a derived from LLC triggers the proliferation of MDSCs.^228^ The expansion and activation of MDSCs induced by glioma‐derived exosomes (GDEs) were facilitated by the hypoxia‐inducible expression of miR‐10a and miR‐21 in GDEs.^229^ This evidence supports the hypothesis that TDEVs can affect the proliferation, differentiation, and immunosuppressive properties of MDSCs, revealing the significant role of EVs in MDSCs.

#### 3.5.3 Macrophages

Widespread stimulation of M2 macrophage polarization to modulate the immune‐suppressing microenvironment by EVs has been observed. Exosomal circZNF451 stimulates an M2 phenotype through improving the degradation of FXR1 by TRIM56, which in turn activates the ELF4‐IRF4 pathway.^230^ After macrophages internalized miR‐145, which was secreted by CRC cells via EVs, histone deacetylase 11 was downregulated to mediate M2 polarization.^231^ Multiple studies have also implicated EVs originating from PC cells as the cause of M2 macrophage polarization.^232^, ^233^, ^234^ CD39 expression in macrophages is increased by HCC‐derived exosomal circTMEM181, which aids in immunosuppression and anti‐PD‐1 resistance. As a result, resistance to anti‐PD‐1 therapy in HCC can be rescued by inhibiting the ATP–adenosine pathway via CD39 targeting macrophages.^235^ Research has revealed how Golgi membrane protein 1 (GOLM1) inhibits CD8+ T cells by transferring PD‐L1 to TAMs through exosome‐dependent mechanisms. By suppressing the expression of Rab27b, GOLM1 enhances the transport of PD‐L1 into exosomes and facilitates PD‐L1 deubiquitination in HCC cells.^236^ HCC cells release LOXL4 protein rather than mRNA, which is mostly internalized by hepatic macrophages through exosomes, resulting in sustained T‐cell exhaustion and tumor progression.^237^ Notably, PD‐L1 expression in TAMs has been observed to be increased by EVs. Endoplasmic reticulum stress in breast cancer increases exosomal miR‐27a‐3p expression and facilitates macrophage PD‐L1 expression, which mediates immune evasion.^238^ N‐acetyl‐l‐cysteine neutralization of ROS increases miR‐155‐5p in exosomes that are internalized by macrophages; this results in PD‐L1 downregulation and a decrease in macrophage infiltration, both of which are indicative of T‐cell activation.^239^ The study of OSCC has focused on Epstein–Barr virus‐encoded small RNAs (EBER‐1), which can influence neighboring immune cells via exosome transfer. Exosomes carrying EBER‐1 stimulated the production of IL‐6, TNF‐α, and indoleamine‐2,3‐dioxygenase (IDO) in macrophages in vitro. The cytolytic activity of CD8+ T cells was reduced by the EBER‐1‐activated IDO in macrophages.^240^ TAMs serve as intermediaries in the intracellular communication between tumors and other stromal cells, facilitating the receipt of signals from EVs and their subsequent delivery to other cells.

#### 3.5.4 Other immune cells

NK cells have gained considerable attention in the field of cancer therapy due to their formidable antitumor capabilities. NK cells experience fatigue due to the downregulation of IFN and TNF‐α secretion. Mechanistically, exosomal circUHRF1 secreted by HCC exerts its function to upregulate TIM‐3 expression via miR‐449c‐5p sponging when delivered to NK cells.^241^ Neutrophils can influence the development of tumors through immunological regulatory functions as well. In breast cancer, Lin28B facilitates metastasis to the lungs through recruiting neutrophils and promoting N2 neutrophil conversion by creating an immune‐suppressive premetastatic niche. Furthermore, low exosomal let‐7s generated by breast cancer cells are necessary for Lin28B‐induced immune suppression.^242^

In general, the data outlined above establish a potential correlation between EVs discharged by neoplastic cells and alterations in the bioactivity of immune cells. The comprehension of these mechanisms is imperative to effectively utilize the immune system as a means to conquer cancer.

## 4 EVs AS BIOMARKERS IN CANCER DIAGNOSIS AND PROGNOSIS

EVs have great potential as sensitive and precise markers for comprehending the mechanisms underlying the emergence of disease processes. While circulating tumor DNA (ctDNA) is another critical component in cancer diagnostics and prognostics, EVs provide numerous advantages over ctDNA. First, the EVs comprise RNA, which contributes to an increase in the number of mutant copies that are available for sampling in comparison with ctDNA.^243^ mRNAs in EVs are actively released rather than being passively expelled from necrotic or apoptotic cells like ctDNA.^244^ These EVs are easily identifiable through electron microscopy due to their uniform size.^245^ Furthermore, the lipid bilayer protects EV cargos, preventing their degradation and rendering them suitable for study.^246^ Actually, the utilization of EVs in conjunction with cfDNA should demonstrate greater potential in accurately determining tumor origin.^247^

### 4.1 EV‐associated biomarkers for early cancer detection

Distinct cargo such as proteins, mRNAs, or metabolic profiles are shown in EVs comparing early‐stage cancer patients to healthy controls. Given that the level of several proteins was higher in EVs isolated from the plasma or serum of tumor patients, they have been suggested as a potential biomarker for tumor diagnosis (Table 1).^248^, ^249^, ^250^ In addition to plasma/serum, higher levels of associated proteins were observed in EVs in cerebrospinal fluid from brain tumors,^251^ urine from balder cancer^252^ and renal cell carcinoma,^253^ ascites from ovarian cancer,^254^ and tissue from the primary tumor site.^251^, ^254^ Even though protein‐based biomarkers have gained a lot of attraction among EV biomarkers, developing them becomes more difficult when dealing with complicated samples such as serum or plasma. These samples contain a lot of nonvesicular proteins, which makes it difficult to isolate low‐abundance protein complexes. Additionally, heterogeneous posttranslational modification adds another layer of complexity to these samples.^255^

**TABLE 1:** Extracellular vesicle cargos as diagnostic and prognostic biomarkers.

Categories	Cargos	Tumors	Origin	Functions	References
Proteins	EGFRvIII and TGF‐β	Brain cancer	Serum/plasma	Diagnosis and prognosis	^256^
	ARG1, CD3, PD‐L1, PD‐L2	Gastric cancer	Serum/plasma	Diagnosis and prognosis	^257^
	Del‐1	Breast cancer	Serum/plasma	Diagnosis	^248^
	IAP4, PSA, GGT1, ITGA2	Prostate cancer	Serum/plasma	Diagnosis and prognosis	^258^, ^259^, ^260^, ^261^
	GPC1, EphA2	Pancreatic cancer	Serum/plasma	Diagnosis and prognosis	^250^, ^262^
	TYRP2, VLA‐4, HSP70, HSP90, MET, S100B, MIA, CD63, Caveolin	Melanoma	Serum/plasma	Diagnosis and prognosis	^28^, ^249^, ^263^
	CD91	Lung cancer	Serum/plasma	Diagnosis	^264^, ^265^
	LG3BP, PIGR	Cholangiocarcinoma	Serum/plasma	Diagnosis	^266^
	GPC1, CD147, CPNE3, TSPAN1	Colorectal cancer	Serum/plasma	Diagnosis and prognosis	^267^, ^268^, ^269^, ^270^
	IL13Rα2	Brain cancer	Cerebrospinal fluid	Diagnosis	^251^
	EpCAM, CD24, CA125	Ovarian cancer	Ascites, tissue culture	Diagnosis	^254^
	TACSTD2	Bladder cancer	Urine	Diagnosis	^252^
	CAIX, MMP‐9, DKK4, CP, PODXL, EMMPRIN	Renal cell carcinoma	Urine	Diagnosis	^253^
miRNA	miR‐1246, miR‐21, miR‐105, miR‐27a, miR‐155, miR‐376a, miR‐376c	Breast cancer	Serum/plasma	Diagnosis and prognosis	^271^, ^272^, ^273^, ^274^
	miR‐125a‐3p, miR‐19a, miR‐92a, let‐7a, miR‐1224‐5p, miR‐1229, miR‐1246, miR‐150, miR‐21, miR‐223 miR‐23a	Colorectal cancer	Serum/plasma	Diagnosis and prognosis	^275^, ^276^, ^277^
	miR‐320, miR‐574‐3p	Glioblastoma	Serum/plasma	Diagnosis	^278^
	miR‐1247‐3p, miR‐18a, miR‐221, miR‐222, miR‐224	Hepatocellular carcinoma	Serum/plasma	Diagnosis and prognosis	^102^, ^279^
	miR‐375, miR‐141, miR‐1290, miR‐375, miR‐6068, miR‐1915‐3p, miR‐6716‐5p, miR‐3692‐3p,	Prostate cancer	Serum/plasma	Diagnosis and prognosis	^62^, ^280^, ^281^
	miR‐1246, miR‐4644, miR‐3976, miR‐4306, miR‐17‐5p, miR‐21, miR‐10b, miR‐10b, miR‐21, miR‐30c, miR‐181a, miR‐let7a	Pancreatic cancer	Serum/plasma	Diagnosis and prognosis	^282^, ^283^, ^284^, ^285^
	let‐7b‐5p, let‐7e‐5p, miR‐21‐5p, miR‐24‐3p, miR‐151a‐5p, miR‐30a‐3p, miR‐200b‐5p, miR‐629, miR‐100, miR‐154‐3p	Lung cancer	Serum/plasma	Diagnosis	^286^, ^287^
	miR‐29a, miR‐25‐3p, miR‐296‐5p, miR‐92a‐3p, miR‐5189‐3p, miR‐16‐2‐3p, miR‐223‐5p, miR‐346, miR‐34a‐5p	Papillary thyroid carcinoma	Serum/plasma	Diagnosis and prognosis	^288^, ^289^, ^290^, ^291^, ^292^
	miR‐21	Glioblastoma	Cerebrospinal fluid	Diagnosis	^293^
	miR‐21	Ovarian cancer	Peritoneal fluid	Prognosis	^294^
	miR‐30q‐5p	Ovarian cancer	Urine	Diagnosis	^295^
mRNA	EGFRvIII mRNA	Glioblastoma	Serum/plasma	Diagnosis	^94^
	hTERT mRNA	Pan‐cancer	Serum/plasma	Diagnosis	^296^
	MMP1	Ovarian cancer	Ascite	Prognosis	^297^
	AR‐V7	Prostate cancer	Urine	Diagnosis	^298^
DNA	Mutated KRAS, p53 DNA, NOTCH1, BRCA2 DNA	Pancreatic cancer	Serum/plasma	Diagnosis	^299^, ^300^, ^301^
	MLH1, PTEN, and TP53 DNA	Prostate cancer	Serum/plasma	Diagnosis	^302^

Abbreviations: AR‐V7, androgen‐receptor splice variant 7; CAIX, carbonic anhydrase IX; CP, ceruloplasmin; CPNE3, copine 3; Del‐1, fibronectin and developmental endothelial locus‐1; DKK4: Dickkopf‐related protein 4; EGFRvIII, epidermal growth factor receptor variant III; EMMPRIN, extracellular matrix metalloproteinase inducer; EpCAM, epithelial cell adhesion molecule; EphA2, ephrin type‐A receptor 2; GGT1, gamma‐glutamyltransferase 1; GPC1, glypican‐1; HSP, heat shock protein; hTERT, human telomerase reverse transcriptase; IAP4, survivin; IL13Rα2, interleukin 13 receptor subunit alpha 2; ITGA2, integrin subunit alpha 2; LG3BP, galectin‐3‐binding protein; MET, proto‐oncogene c‐Met; MIA, melanoma inhibitory activity; MMP, matrix metalloproteinase; PIGR, polymeric immunoglobulin receptor; PODXL, podocalyxin; PSA, prostate‐specific antigen; S100B, S100 calcium‐binding protein B; TACSTD2, tumor‐associated calcium signal transducer 2; TSPAN1, tetraspanin 1; TYRP2, tyrosinase‐related protein‐2; VLA‐4, very late antigen 4.

Table 1 provides a summary of the EV‐associated nucleic acids (mRNA, miRNA, DNA) that have been detected in body fluids for early tumor detection.^303^ The yield, purity, stability, and particularly the RNA content of EVs may be impacted by the RNA separation technique used. Thus, choosing the RNA separation technique based on the study's design and the body fluids’ accessibility is crucial.^304^ There is a great deal of interest in exploiting the DNA found in circulating EVs as liquid biopsies since it has been demonstrated that EVs contain transposable elements, including ssDNA, mtDNA, and genomic DNA (gDNA).^305^ When compared with DNA from cells without membranes, EVs have a higher concentration of tumor DNA since the defense against DNases keeps the DNA confined in the EV membranes relatively stable.^306^ Furthermore, EV DNA's short half‐life allows for a precise depiction of the dynamic tumor hallmark, making it a valuable instrument for tracking the advancement of tumors over time and how they react to antitumor therapy.^301^

Lipids and metabolites in EVs have demonstrated a growing amount of promise as biomarkers in tumor detection with the advancement of lipidomics and metabolomics. Several types of studies, including PC and pancreatic cancer studies, have involved exosomal metabolomic or lipidomic profiling, which might be used to obtain unique biomarker data.^307^ To identify PC biomarkers with enhanced sensitivity and specificity, Clos‐Garcia et al.^308^ reported that a thorough examination of the composition of urine EVs may provide a window of opportunity. Consistent with the probable increase in androgen production, these authors detected elevated quantities of steroid hormones in urinary EVs, which supports the noninvasive use of urine EVs to provide information on metabolic changes in malignant tissue.^308^ A study reported that the amount of lipoprotein lipase (LPL) in EVs produced from ovarian cancer cells was shown to be considerably higher than in ovarian surface epithelial cells, indicating that LPL may be useful in the early detection of ovarian cancer.^309^ Researchers used liquid chromatography–mass spectrometry (LC–MS) to examine the lipidomic profile of exosomes formed from CRC cell lines and patients. According to the findings, there were notable differences in the lipidomic signature between exosomes derived from nonmetastatic and metastatic cell lines and patient plasma, especially in the case of glycerophospholipids and sphingolipids.^310^ These results offer important perspectives on the possible use of clusters of lipid biomarkers instead of single molecules for the diagnosis of CRC. There is much need for new biomarkers for HCC surveillance in cirrhotic patients. Several modifications in the lipid content of exosomes linked to HCC have been explored to indicate the changes in ferroptosis, retrograde endocannabinoid signaling, and glycerophospholipid metabolism.^311^ To sum up, this study found altered pathways in exosomes that might facilitate the growth and progression of tumors, as well as potential biomarkers for the early detection of HCC. Ketone body metabolism and FAO show distinct signatures in patient serum‐derived EVs, according to a comparison of healthy controls and patients receiving radiation therapy for head and neck cancer.^88^, ^312^ These illustrations demonstrate the significance of lipids and metabolites found in EVs for the potential of detecting early tumors and guiding therapy approaches.

Artificial intelligence (AI) has been a popular choice for various cancers' early detection. Within AI, machine learning (ML) is a subfield that learns from large amounts of data and uses algorithms to evaluate in order to create models that support decision‐making and prediction. Through the analysis of exosome surface‐enhanced Raman spectroscopic profiles, Shin et al.^313^ employed AI to detect six types of solid tumors much earlier, while another study used a panel of exosome‐associated proteins calculated by ML to distinguish between different kinds of tumors.^314^ To quantitatively analyze the pleiotropic impact of EVs, Nagrath et al.^315^ performed a novel computational methodology of the contribution of metabolite cargo to the metabolism of cancer cells delivered from CAFs, known as exosome‐mediated metabolic flux analysis (Exo‐MFA), based on an examination of the flux of 13C metabolic products. Exo‐MFA is capable of predicting the rate of exosome internalization and determining the contribution of exosomal cargo to metabolites inside PDAC cells, and the results of this analysis strongly suggested that exosome‐supplied metabolites can assist in PDAC metabolism during the initial phases of nutrient restriction.^315^ Additionally, predictive panels were generated using an ML algorithm, which was also capable of distinguishing tumors from healthy controls.^316^ In an effort to identify mutated proteins in circulating exosomal cargo, Kim et al.^317^ implemented a deep learning algorithm and nanoplasmonic spectra. Different mutant variants of EGFR were identified in the blood of lung cancer patients using this model. Actually, confounding factors in cancer samples and data acquired from various centers should be taken into account by ML models in order to enhance cancer prediction.

### 4.2 EV cargo profiling for predicting treatment response and disease progression

In addition to their diagnostic potential, EVs also provide insights into the whole spectrum of cancer development, from the disease's earliest beginnings to metastases and treatment responses. An investigation revealed that the presence of Tim‐3 and Galectin‐9 proteins in EVs was linked to age, distant metastasis, and TNM staging in individuals with NSCLC.^318^ EV cargos like NY‐ESO‐1, EGFR, PLAP, EpCam, and Alix had a substantial effect on overall survival (OS), which depended on their concentration in NSCLC.^265^ Hoshino et al.^56^ have identified a set of tumor‐type‐specific EV‐associated proteins in both tumor tissues and plasma, which can be used to accurately diagnose cancers of unknown origin. Furthermore, EV‐mRNA can serve as a biomarker for prognosticating the impact of ICIs. Del et al.^319^ conducted a study to find the relationship between the effectiveness of anti‐PD‐L1 immunotherapy and the quantity of EV‐mRNA PD‐L1 expressed in plasma. Amounts of studies suggested that certain immunotherapy patients—many of whom exhibit no response at all or continue to progress their disease—had rising exosomal PD‐L1 levels over time. Finally, increased exosomal PD‐L1 levels may result in poor efficacy both during and after treatment.^153^, ^320^, ^321^ Currently, numerous studies are endeavoring to address the issue of immunotherapy resistance by targeting exosomal PD‐L1 and related modulators.^235^, ^322^

Tumor‐specific biomarkers have the potential to forecast the effectiveness of treatment and tumor progression. Compared with traditional biomarkers like CA199 and CEA, HOTTIP in serum EVs demonstrated superior accuracy in GC.^323^ Urinary EVs lncRNA PCAT‐1 and MALAT1 overexpression in bladder cancer were linked to a poor recurrence‐free survival rate and facilitated recurrence prediction.^324^ Higher circulating levels of EV lncRNA‐ATB in HCC were linked to decreased OS and progression‐free survival (PFS),^325^ while uroepithelial carcinoma patients may be at risk of metastasis if they have increased levels of circRNA PRMT5 in the serum and urine.^326^ Although the initial analysis of EV cargo focuses more on mRNA, miRNA and lncRNA, subsequent research has uncovered a plethora of other forms of ncRNAs, such as tRNA and tRNA fragments, Y RNA, piRNA, and rRNA, which may function as biomarkers for therapy response.^327^ Using LC–MS‐based untargeted lipidomics, Tao et al.^328^ sought to identify potential metabolic biomarkers linked to tumor stage, CA199, CA242, and tumor diameter in pancreatic cancer. A plasma exosome‐based metabolome marker pattern from a random forest model that can predict the recurrence of esophageal squamous cell carcinoma revealed a significant increase in palmitoleic acid in recrudescent individuals.^329^ The levels of metabolites carried in serum‐derived exosomes for glycolysis, gluconeogenesis, the tricarboxylic acid cycle, pyruvate metabolism, and the mitochondrial electron transport chain were shown to be significantly different in head and neck cancer patients before and after radiotherapy, which mirrored putative radiotherapy‐induced alterations in a variety of metabolic pathways.^88^ In the end, the capacity to use EVs as dynamic markers of cancer development holds the potential to completely redefine cancer treatment, resulting in more accurate and effective therapeutic strategies.

### 4.3 Diagnostic potential of EVs in liquid biopsies and circulating tumor cells

EVs may prove to be a valuable noninvasive diagnostic and screening instrument in the early stages of the disease. Taking circulating EV proteins for example, pancreatic cancer patients can be distinguished from healthy donors using GPC1‐positive circulating exosomes according to the study of Melo et al.^262^ A study indicates that the decrease in gastrokine 1 protein in serum EVs could serve as a valuable diagnostic for identifying individuals with GC.^330^ Additionally, it has been claimed that novel diagnostic biomarkers for breast cancer have been discovered in the form of phosphorylated proteins from the plasma EVs of patients.^331^ Concurrently, it has been discovered that miRNAs from EVs may serve as potential diagnostic indicators for many malignancies, such as ovarian,^332^ hepatocellular,^279^ and breast cancers.^333^ A cluster of miRNAs identified using EV small RNA sequencing in both the ascites and plasma was found to exhibit a high level of diagnostic accuracy.^334^ Jin et al.^286^ conducted a study where they used miR‐23a‐3p, miR‐486‐5p, let‐7b‐5p, and let‐7e‐5p in exosomes to diagnose NSCLC at an early stage. The study achieved an AUC value of 0.899, a sensitivity of 80.25%, and a specificity of 92.31%.^286^ And the diagnostic efficacy of early‐stage tumors can be enhanced by the combination of exosomes and serum markers. Based on plasma exosomes miR‐15a‐5p, the AUC for the diagnosis of stage I endometrial cancer was only 0.813. However, the AUC was increased to 0.899 when CEA and CA125 were included in the analysis.^335^ Another study discovered that the combination of 8exo‐miRNA and 5cf‐miRNA, along with CA199, effectively distinguished patients with tumors that had a CA199 level below 37 U/mL. This identification had an AUC value of 0.99.^336^ The comprehensive range of diagnostic capabilities of EVs in liquid biopsies and circulating tumor cells (CTCs) has been documented in Table 1. While EVs show promise for early tumor diagnosis, there are still constraints that need to be addressed in order to enhance the accuracy and dependability of EVs.

## 5 THERAPEUTIC TARGETING OF EVS IN CANCER

### 5.1 Strategies to inhibit EV biogenesis and secretion

The majority of present treatment approaches aim to reduce EV production, secretion, and uptake, impede EV‐mediated intercellular communication, and eliminate a particular active molecule found in EVs (as shown in Table 2).

**TABLE 2:** Commonly used agents for inhibiting extracellular vesicle biogenesis and secretion.

Categories	Drugs	Biological functions	References
Lipid metabolism	Pantethine	Reduction of total cholesterol level	^337^
	Imipramine	Inhibition of aSMase	^338^
	GW4869	Inhibition of nSMase	^127^, ^339^, ^340^, ^341^, ^342^
	Spiroepoxide	Inhibition of nSMase	^343^
	DPTIP	Inhibition of nSMase	^344^
	Glibenclamide	Reduction of cholesterol level	^345^
	Indomethacin	Inhibition of ABCA3, an intracellular protein involved in lipid transport	^346^
Cytoskeleton remodeling	Calpeptin	Calpain inhibitor	^347^
	Y‐27632	ROCK1 and ROCK2 inhibitor	^348^
	Cytochalasin D	Inhibition of actin polymerization	^349^
	U0126	MEK 1 and MEK 2 inhibitor	^350^
	NSC23766	Rac1 inhibitor	^351^
	Manumycin A	Inhibition of farnesyltransferases	^352^
	Tipifarnib	Inhibition of farnesyltransferases	^353^
	Chloramidine	Inactivation of PAD enzymes	^354^
	BIM‐1	PKC inhibitor	^355^
	Sulphisoxazole	Endothelin receptor A	^356^
	Macitentan	Endothelin receptor A	^322^
	SyntOFF	Syndecan–syntenin–alix pathway	^357^
Calcium regulation	Ketotifen	Preventing the influx of calcium into cells	^358^
	Dimethyl amiloride	Inhibition of H^+^/Na^+^ and the Na^+^/Ca^2+^ channels	^359^
Exosomes uptake	Free heparan sulfate chain	Targeting cell‐surface heparan sulphate proteoglycans	^360^
	Dynasore	Endocytosis inhibitor	^361^
	Methyl‐β‐cyclodextrin	Remove cholesterol from membranes and interfere lipid rafts stability	^362^
	Gefitinib	Inhibition of nonclassical endocytosis pathway	^363^

Abbreviations: aSMase, acid sphingomyelinase; nSMase, neutral sphingomyelinase; PAD, peptidyl‐arginine deiminases; PKC, protein kinase C; ESCRT, endosomal sorting complex required for transport; BIM‐1, bisyndoylmaleimide 1.

In terms of exosomes, mechanisms that produce or modify cell membrane asymmetry seem to be crucial for the formation of MVs. In actuality, cholesterol appears to play an important role in the budding of the cell membrane, as lipid rafts are essential for this process and their reduction decreases the release of MVs.^364^, ^365^ Pantethine may be employed to impede the discharge of MVs, which has the ability to impede the translocation of PS on the outer membrane leaflet, a crucial mechanism involved in the generation of EVs.^366^ Doxorubicin (Dox)‐resistant cells generated more MVs upon stimulation with the agonist A23187, but Dox‐sensitive MCF‐7 cells did not. After receiving pantethine as a pretreatment, this effect was lessened, resulting in a 24% total MV reduction.^337^ Therefore, in order to gain a comprehensive understanding of pantethine and its potential as a selective inhibitor of MVs, it is necessary to expand upon the previous work. The tricyclic antidepressant imipramine has gained notice for its ability to inhibit the enzyme acid sphingomyelinase (aSMase). Since it improves membrane fluidity, exosome release, and EV synthesis, aSMase enzymes catalyze the hydrolysis of sphingomyelin to ceramide, a process engaged in the formation of both exosomes and MVs.^337^ It should be noted that while imipramine was shown to disrupt exosomes and MVs in PC‐3, these vesicle types were not examined individually including EVs of both <150 and >150 nm‐sized vesicles.^338^ It would be beneficial to conduct more comprehensive investigations of imipramine at subtoxic concentrations and to conduct a thorough characterization of the EVs.

GW4869, a neutral sphingomyelinase (nSMase) inhibitor, is the most extensively used pharmaceutical treatment for preventing exosome production. GW4869 has proven to be essential for immunological modulation due to its ability to facilitate the suppression of exosomes containing PD‐L1, which rejuvenated T cells and boosted ferroptosis.^340^ Besides, the oxygen consumption rate in breast cancer cells could be partially restored by GW4869, demonstrating that exosomes released by CAFs were responsible for the decrease in mitochondrial respiratory function in tumor cells and could be targeted to deter tumor metabolism.^127^ Deterred antitumor immunity could be alleviated by reducing the suppressive effects of exosomes. A set of semiconductor polymers encapsulating an exosome inhibitor GW4869 and ferroptosis inducer was utilized to fabricate phototheranostic metal‐phenolic networks (PFG MPNs), which were used to alleviate exosomal silencing during DC maturation by augmenting immunogenic cell death.^340^ The fibroblast‐derived conditioned medium enhanced the percentage and clonogenicity of CSCs. Treatment with GW4869, a compound that inhibits exosome production, resulted in a reduction of CSCs and increased susceptibility to 5‐fluorouracil (5‐Fu) or OXA.^339^ In NOD/SCID mice carrying ASPC1 and CAFs, the tumor's growth rate was significantly slower 10 days after cotreatment of gemcitabine and GW4869 than it was in the gemcitabine‐only group. Although GW4869 has been investigated very thoroughly and shown to suppress exosomes, some studies have not described the exosomes’ characterization or their separation process. By targeting nSMase, more drugs like spiroepoxide and DPTIP have been explored to limit exosome secretion by preventing ceramide‐modulated inward budding of MVBs and the consequent release from MVBs.^343^, ^344^ Apart from that, antidiabetic drugs (Glibenclamide)^345^ and anti‐inflammatory drugs (Indomethacin)^346^ are implicated in lipid transport. These compounds have the potential to serve as inhibitors of exosome release, as lipids are fundamental to the formation of MV and exosomes.

MVBs can, as previously indicated, fuse with lysosomes or release exosomes through fusion with the cell membrane. Many proteins interact with actin and microtubules of the cytoskeleton to govern their translocation toward the cell membrane. It appears that calpains can encourage MVs shedding because of their involvement in cytoskeleton remodeling. Therefore, it has been observed that blocking them with calpain inhibitors lowers the number of MVs released from cells.^367^ Calpeptin is the most extensively researched inhibitor of calpain, which has been widely utilized as an MV inhibitor. Calpeptin has been reported to ameliorate MV‐mediated anticancer drug resistance. Jorfi et al.^347^ showed that the accumulation of methotrexate and docetaxel within the cells was made possible by calpeptin's inhibition of MVs release. This accumulation led to a significant reduction in cell proliferation and an increase in cell death compared with the conditions without calpeptin treatment in vitro and in vivo of PC.^347^ Nevertheless, given the positive results of the calpeptin experiments that have been published thus far, more research on the substance's capacity to prevent EV release is necessary. ROCK1 and ROCK2 play a part in the formation of EVs, which could be inhibited by Y‐27632, a selective, reversible, competitive kinase‐activity inhibitor.^348^, ^368^ Mechanistically, ROCK1 and ROCK2 govern actin cytoskeletal remodeling and actomyosin contraction by activating adducin, which keeps the actin network together.^13^ Cerione et al.^348^ explored that ras homolog family member A (RhoA) knockdown prevented the release of EVs from cancer cells. It is intriguing that the presence of EVs along their membrane was eliminated by treatment with Y‐27632, which confirmed the role of Rho‐GTPases in the formation of EVs.^348^ Furthermore, the activation of ERK, which is required for microvesiculation, can be effectively prevented by protein kinase inhibitors such as MEK 1 and MEK 2 inhibitor U0126.^350^ Inhibition of Rac1, a member of the Rho family has been shown to exert exosomal targeting.^351^ As a powerful and selective inhibitor of Ras farnesyltransferases, manumycin A, has been studied as an exosome secretion inhibitor due to Ras's role in exosome release. The biogenesis of exosomes that are dependent on ESCRT was specifically inhibited by manumycin A, as reported by Datta et al.^352^ The production of exosomes was reduced by manumycin A in PC cell lines, with the effect being even more pronounced when manumycin A was used in conjunction with GW4869.^352^ The drug Tipifarnib is another example that employs this comparable mechanism to prevent the discharge of EVs.^353^ A family of Ca^2+^‐dependent enzymes known as the peptidylarginine deiminases (PADs) is responsible for protein deimination. When PAD enzyme activity was pharmacologically inhibited with chloramidine, significantly no deimination of cytoskeletal actin and then less MV release occurred.^354^ Another primary regulator of the mechanism that drives the release of EVs is the calcium‐mediated activation of protein kinase C (PKC) and the externalization of PS.^13^ A study showed that treating PC‐3 cell lines with bisindolylmaleimide‐1 (BIM‐1), a cell‐permeable and reversible PKC inhibitor, reduced EV release by 75%.^355^ Reduced PS externalization has been suggested as the mechanism by which BIM‐1 lowers EV secretion levels. Sulfisoxazole^356^ and macitentan,^322^ two endothelin A receptor antagonists, have recently become available as novel treatments for preventing exosome secretion, inhibiting several Rab proteins as well as elements of the ESCRT‐dependent pathway, such as ALIX and VPS4B. The syndecan‐syntenin‐ALIX pathway, which is associated with exosome biogenesis, is disrupted by SyntOFF through binding to the PDZ domain of syntenin. In breast cancer patients, this perturbation leads to a reduction in proliferation and metastasis, as well as a decrease in exosome secretion.^357^

Given the complexity of the EV release mechanism, different drugs acting on various targets involved in the same signal cascade can impede the same production pathway. Studies indicate that Ca^2+^ is an essential element in the process of Ca^2+^‐dependent membrane fusion and exosome production.^369^, ^370^ Store‐operated calcium channel blockers including ketotifen, an antihistamine reduced the release of exosomes from a variety of cancer cells by 70% through preventing the influx of calcium into cells.^358^ A derivative of amiloride, dimethyl amiloride is a drug used to treat high blood pressure and has been suggested as a potential exosome blocker because of its role of blocking the H^+^/Na^+^ and the Na^+^/Ca^2+^ channels.^359^

Exosome absorption in the receiving cell occurs in a nonrandom manner in conjugation with transmembrane proteins. Several mechanisms, including phagocytosis, macropinocytosis, caveolin‐dependent endocytosis, and clathrin‐mediated endocytosis (CME), can lead to the process.^371^ All in all, the potential of these drugs (such as free heparan sulfate chain, dynasore, methyl‐β‐cyclodextrin, and gefitinib) to impede the uptake of EVs has been examined as shown in Table 2.^360^, ^361^, ^362^, ^363^ These results imply that focusing on different stages of exosome biosynthesis, secretion and uptake may be beneficial for the exploration of efficient drugs.

### 5.2 Utilizing EVs as drug delivery vehicles for targeted therapy

**FIGURE 4:**
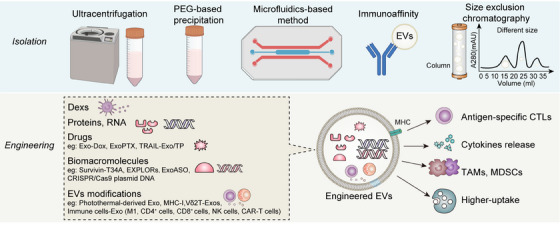
EVs isolation and engineering methods. Diverse approaches utilizing size, density, and surface proteins have been developed for the extraction and enrichment of EVs from complicated biofluids. To improve the delivery ability of isolated EVs, bioengineering methods modify natural EVs by increasing their drug‐loading capacity, targeting accuracy, and resistance to clearance, hence significantly expanding their therapeutic potential. Postengineering, EVs enhance the infiltration of antigen‐specific CTLs, the release of cytokines, and the cellular absorption efficiency of EVs while reducing TAMs and MDSCs. EVs, extracellular vesicles; CTLs, cytotoxic T lymphocytes; TAMs, tumor‐associated macrophages; MDSCs, myeloid‐derived suppressor cells.

It is possible for EVs to avoid being taken up by macrophages, prolong their stay in the bloodstream, and pass through the extracellular matrix and vascular walls. Moreover, EVs possess low immunogenicity and good biocompatibility, enabling their stability and widespread distribution in biological liquids.^21^ Apart from that, they can also enrich the tumor, penetrate the tumor cells, and release carried drugs. The utilization of natural or gene‐engineered EVs as drug‐delivery vehicles offers special advantages because of their diverse biological features (Figure 4).^372^

Since many anticancer chemotherapeutic drugs target intracellular targets, their ability requires them to traverse through cell membranes. The drugs that are loaded in EVs are safeguarded by the lipid bilayer of EVs, which facilitates their entry into cells by interacting with the recipient cells through membrane proteins. Their minimal immunogenicity and toxicity are the most significant advantages of EVs, as they can significantly avoid immune system clearance.^373^ To enhance the selectivity of Dox toward tumor tissues rather than cardiomyocytes, Wei et al.^374^ coincubated Dox with BM‐MSC‐exosomes to create BM‐MSC‐derived exosome‐loaded Dox (Exo–Dox) and showed noticeably higher cellular absorption efficiency and anticancer effects compared with free Dox. In addition, it also showed lower uptake efficiency and toxic effects in cardiomyocytes.^374^ They also indicated that Exo–Dox may accumulate in the tumor site in vivo, attracting osteosarcoma cells via the SDF1–CXCR4 axis. When comparing the Exo–Dox group to the free Dox group, there was a significant decrease in both Ki67‐positive cells and cardiotoxicity.^375^ The existence of MDR decreases the tumor response rate to treatment using traditional chemotherapy drugs. Paclitaxel (PTX) loaded into sonication‐treated exosomes demonstrated significant loading and prolonged drug release. PTX‐loaded exosomes (exoPTX) exhibited notable accumulation within tumor cells and demonstrated an increase in cytotoxicity toward drug‐resistant tumor cells. In vivo, exoPTX also displayed colocalization with the tumor and further inhibition in a pulmonary‐metastasis mouse model of LLC.^373^ After genetic modification of human primary CD8+ T‐cell‐derived exosomes, they were impregnated with IL‐2 and cetuximab (CTX), an anti‐EGFR antibody, which has the potential to augment tumor cytotoxicity and refine cancer targeting.^376^ These suggest that EVs have significant potential for the delivery of therapeutic agents in the treatment of drug‐resistant malignancies, as they facilitate in vivo targeting and enhance the antitumor effects.

Apoptosis is induced by TNF‐related apoptosis‐inducing ligand (TRAIL) in cancer cells. Unfortunately, because of their limited targeting and short half‐life in vivo, TRAIL‐based drugs, such as recombinant human soluble TRAIL, have not yet shown adequate therapeutic efficacy in clinical settings.^377^ Triptolide (TP) was loaded into exosomes released from TRAIL‐overexpressing macrophages Raw264.7 to form TP‐based TRAIL‐engineered exosomes (TRAIL–Exo/TP). In vitro and vivo experiments revealed that TRAIL–Exo/TP significantly inhibited the development and promoted apoptosis of melanoma, in comparison with free TP and TP‐loaded exosomes alone. On the other hand, TRAIL–Exo/TP was found to be biologically safe and did not induce systemic toxicity or myelosuppression.^378^ Exosomes containing TRAIL have been demonstrated in other recent preclinical investigations to cause apoptosis while preventing the growth of cancer in vitro as well.^379^, ^380^ To effectively deliver TP into the tumor, Gu et al.^381^ found another method using arginine–glycine–aspartate (cRGD)‐modified exosomes. This showed strong tumor targeting and a longer half‐life of exosome‐delivered drugs, which reduced systemic toxicity.

In contrast to small‐molecule medicines, degradation occurs in biomacromolecules such as proteins, peptides, and nucleic acids in vivo. Additionally, they would contend with a number of biological obstacles to cell‐to‐cell communication, including cell membranes and endosomes, which restrict the use of biomolecules in anticancer therapy.^382^ Exosomes carrying the biomolecules are highly advantageous for the delivery of biomolecular therapeutics such as exosomes loaded with Survivin‐T34A.^382^ It enhanced tumor cell apoptosis when treating pancreatic cancer cells in conjunction with gemcitabine through inhibiting survivin in tumor cells. Choi et al.^383^ explored a novel method for delivering target proteins within cells using optically reversible protein–protein interactions (EXPLORs). It has been demonstrated that treating recipient cells with protein‐loaded EXPLORs greatly increases the intracellular levels of cargo proteins.^383^ Delivery of nucleic acid drugs in exosomes such as antisense oligonucleotides (ASOs) has been demonstrated to significantly decrease the target gene expression.^384^ Significant anticancer activity is produced by exosomes loaded with the ASO of C/EBPβ or STAT6 (exoASO), which transforms M2 into M1 macrophages.^383^ CRISPR/Cas9 has been widely used in genome editing based on common viral vectors or nonviral vectors. Both vectors have drawbacks like immunogenicity and insertional mutagenesis, organ toxicity and low biocompatibility respectively.^385^ Exosomes offer a promising substitute for CRISPR/Cas9 gene delivery methods. To overcome these disadvantages, McAndrews et al.^386^ demonstrated that exosomes carrying CRISPR/Cas9 plasmid DNA may specifically target pancreatic cancer cells with mutant KRAS G12D, leading to the deletion of target genes and the subsequent inhibition of tumor growth. Hao et al.^387^ reported that Erastin (iron ion inducers) and RB (photosensitizers) were successfully enclosed within exosomes through the process of sonication, which exhibited a potent induction of ferroptosis in tumors following exposure to radiation. To take advantage of the killing effect that the zinc oxide nanocrystal cargo conveys against lymphoma cells, EVs that are engineered from healthy cells and loaded with inorganic nanoparticles and monoclonal antibodies can be triggered on‐demand by an external stimulus.^388^ The recently developed iRGD‐tagged exosomes expressing EBV–miR‐BART1‐5p are a potentially useful tool for inhibiting tumor cell‐mediated vasculogenic mimicry and endothelial sprouting angiogenesis in nasopharyngeal carcinoma.^389^ Photothermal and hyperthermia eradicate cancer cells and acquire exosomes containing significantly more immune‐activating molecules in breast cancer. This indicates that thermal treatments, particularly those using photothermal‐derived exosomes, enhance the vulnerability of malignancies to T cells, thereby inhibiting the growth of tumors.^390^ EV drug delivery has gained significant momentum during the past decades, developing into a potentially effective biological therapeutic approach. Modern methodologies allow scientists to precisely target therapy by inserting therapeutic chemicals onto the surface of EVs.

EVs that have been modified through the application of bioengineering techniques in order to improve their drug‐loading efficiency, targeting capability, and resistance to clearance are referred to as engineered EVs.^391^ Exogenous loading methods mean the process of loading pharmaceuticals directly into pre‐separated exosomes by the utilization of membrane penetration methods while endogenous loading methods are based on the parent cell modification through direct transfection and coincubation.^392^, ^393^ However, both of them have disadvantages, including the small size of exosomes limiting their ability to load drugs and the high level of technical skill and equipment support needed for exogenous loading. Moreover, it also faces poor exosome‐storage stability resulting from exosomes’ propensity to aggregate and degrade over extended periods of storage. As for endogenous loading methods, higher costs and the verification of drug‐loading efficacy remain hurdles. It also has trouble choosing the best drugs and donor cells.^394^

### 5.3 Modulating EV‐mediated intercellular communication to enhance treatment responses

Certain cancer patients benefit from extended PFS as a result of ICI‐based treatments; however, a significant proportion of patients experience relapses due to acquired resistance. Consequently, it is critical to clarify the underlying mechanisms and devise approaches to overcome ICI resistance (Table 3).^395^, ^396^ An increasing amount of data currently points to links between poor prognosis, ICI resistance and substantial infiltration of immunosuppressive M2‐like TAMs.^397^, ^398^ In terms of the underlying mechanisms, M2‐derived exosomes can confer ICI resistance by delivering apolipoprotein E to cancer cells, downregulating MHC‐I expression and reducing intrinsic tumor immunogenicity.^399^ To determine whether TAM‐derived exosomes stimulated tumor immune evasion, Jiang et al.^400^ reported that LINC01232 enhanced the transcription of NBR1 by mechanistically binding to E2F2 and facilitating its entry into the nucleus of glioma cells. The expression of MHC‐I on the surface of tumor cells is induced, resulting in the evasion of CD8+ CTL immune attack by tumor cells.^400^ The distinct exosomes that contain BMI1 stimulate the growth and metastasis of CCA via autocrine and paracrine processes. Furthermore, one study showed that by encouraging repressive H2A ubiquitination in CCA cells, BMI1 suppresses chemokines that recruit CD8+ T cells, implicating a potential novel combination therapy of anti‐PD‐1 and BMI1 inhibitors for CCA.^401^ Anti‐PD‐1 treatment sensitivity could also be compromised by exosomal circZHF451 through the induction of M2 macrophage polarization in lung adenocarcinoma (LUAD). These researches elucidated a novel mechanism by which anti‐PD‐1 resistance develops and identified a potential biomarker for predicting anti‐PD‐1 efficacy.^230^

**TABLE 3:** Extracellular vesicles as therapy resistance biomarkers.

Therapy	Biomarkers in EVs	Cancers	Mechanism	References
ICI resistance	ApoE	Gastrointestinal cancer	M2 polarization	^399^
ICI resistance	LINC01232	Glioma	M2 exosomes inhibit CD8+	^400^
ICI resistance	BMI1	CCA	CD8+ T density	^401^
ICI resistance	circZHF451	LUAD	M2 Polarization	^230^
Paclitaxel	gp96	Breast cancer	CD8+ T pyroptosis	^402^
Cisplatin	PD‐L1	NSCLC	CD8+ T inactivation	^208^
Cisplatin	miR‐223	Ovarian cancer	Macrophage‐derived miR‐223–PTEN/PI3K/AKT	^403^
Cisplatin	miR‐21	Pan‐tumor	Macrophage inflammasomes	^404^
Cisplatin	miR‐182	HCC	TP53INPI	^405^
Cisplatin	miR‐100‐5p	Lung cancer	mTOR	^406^
Cisplatin	miR‐423‐5p	Breast cancer	–	^407^
Cisplatin	circ_0008928	NSCLS	circ_0008928–miR‐488–HK2–glycolysis	^408^
Cisplatin	Exosomes	Ovarian cancer	Hypoxia–Rab27a	^184^
Cisplatin	PKM2	NSCLC	Hypoxia–PKM2–glycolysis	^409^
Cisplatin	PKM2	NSCLC	Shikonin–PKM2–glycolysis	^410^
Cisplatin	Gelsolin	Ovarian cancer	HIF‐1α	^411^
Gemcitabine	CHI3L1, FN1	Pancreatic cancer	TAM–EVs	^412^
Gemcitabine	lncRNA UCA1	Pancreatic cancer	lncRNA UCA1–SOCS3/EZH2 axis	^413^
Gemcitabine	circZNF91	Pancreatic cancer	circZNF91–miR‐23b‐3p–SIRT1–HIF‐1α–glycolysis	^414^
Carboplatin	Exosomes	Ovarian cancer	HIF‐1α–glycolysis	^415^
Oxaliplatin	ciRS‐122	Colorectal cancer	ciRS–miR‐122–PKM2 axis–glycolysis	^73^
5‐FU	miR‐101‐3p	Colorectal cancer	miR‐101‐3p–HIPK3–VDAC1–OXPHOS	^138^
5‐FU, oxaliplatin, doxorubicin	circ_0094343	Colorectal cancer	circ_0094343–miR‐766‐5p–TRIM67–glycolysis	^416^
Temozolomide	circ_0072083	Glioma	Shikonin‐circ_0072083–miR‐1252‐5p–ALKBH5–Nanog‐glycolysis	^417^
Temozolomide	miR‐106a‐5p	Glioma	Hypoxia–miR‐106a‐5p–PTEN	^418^
Temozolomide	miR‐30b‐3p	Glioma	Hypoxia–miR‐30b‐3p–RHOB–proliferation	^419^
Melphalan, bortezomib	Acid sphingomyelinase	Multiple myeloma	Acid sphingomyelinase–lipid metabolism	^136^
Osimertinib	Exosomes	NSCLC	M2 polarization	^420^
Gefitinib	DOK3	Lung cancer	Active T cells	^421^
RT resistance	ALDOA, AKDH3A1	NSCLS	ALDOA, ALDH3A1–glycolysis	^422^
RT resistance	ANGPTL4	NSCLC	Hypoxia–ANGPTL4	^423^
RT resistance	miR‐301a	Glioma	miR‐301a–TCEAL7	^424^

Abbreviations: 5‐Fu, 5‐fluorouracil; ApoE, apolipoprotein E; CCA, cholangiocarcinoma; EVs, extracellular vesicles; HCC, hepatocellular carcinoma; ICI, immune checkpoint inhibitor; LUAD, lung adenocarcinoma; NSCLC, non‐small cell lung cancer; OXPHOS, oxidative phosphorylation; PKM2, pyruvate kinase isozymes M2; RT, radiotherapy; TAM, tumor‐associated macrophage.

BC cells acquired PTX resistance in response to gp96 exosomes derived from PTX‐resistance‐BC, whereas CD8+ T cells isolated from human peripheral blood mononuclear cells experienced pyroptotic cell death.^402^ Cisplatin resistance was enhanced in NSCLC cells derived from PD‐L1‐containing exosomes.^208^ By means of exosomal miR‐21 delivery, tumor cells can inhibit the activity of inflammasomes in macrophages in response to chemotherapy in malignancies undergoing snail‐induced EMT.^404^ It has been reported that EVs containing miR‐182 promote HCC resistance to cisplatin by regulating TP53INPI.^405^ A similar transformation of the chemoresistance phenotype has also been reported in cisplatin‐resistant lung and breast cancers by transferring miR‐100‐5p and miR‐423‐5p, respectively.^406^, ^407^ The actin‐associated protein plasma gelsolin can be secreted and transported via EVs from chemoresistant ovarian cancer cells to their chemosensitive counterparts to confer cisplatin resistance.^411^ In addition to PTX and cisplatin, several frequently used chemotherapeutic medicines have been found to exhibit resistance through EV transportation such as gemcitabine,^412^, ^413^, ^414^ carboplatin,^415^ OXA,^73^ 5‐Fu,^138^ Dox,^416^ temozolomide,^417^, ^418^, ^419^ melphalan, and bortezomib.^136^ Taking gemcitabine for example, macrophage‐derived EVs secrete CHI3L1 and FN1 as their most prevalent cargo proteins. Through the ERK signaling pathway, they could play a crucial role in transferring gemcitabine resistance characteristics to cancer cells through intercellular communication in pancreatic cancer.^412^

Molecularly targeted therapies employ various mechanisms to combat cancer, including impeding the growth and division of cancer cells by disrupting signaling pathways, inhibiting the formation of blood vessels that support tumor growth, transferring cytotoxic substances directly to cancer cells, and depriving cancer cells of essential hormones necessary for their growth.^425^ Identifying the mechanism of resistance to targeted therapy and developing strategies to overcome it will be a highly effective approach to tumor treatment. In NSCLC, osimertinib, a third‐generation EGFR‐TKI, can inhibit both sensitive EGFR mutations and acquired EGFR T790M mutations.^426^ While osimertinib has shown considerable effectiveness and acceptable safety, the problem of resistance is still unavoidable. Exosomes originating from osimertinib‐resistant cells were shown to be internalized by macrophages, resulting in macrophage polarization toward the M2 phenotype.^420^ However, there was a significant correlation between the prolongation of PFS following gefitinib treatment and plasma exosomal Docking Protein 3 (DOK3), a molecule implicated in B‐cell receptor signaling in lung cancer. These results imply that patients are more likely to benefit from gefitinib therapy if they have active T‐ and B‐cell immunity.^421^

Radiation therapy is used prevalently as a therapeutic modality for the management of cancer. The intrinsic metabolism of tumors is important for their resistance to ionizing radiation, making radiotherapy less effective. The exosomal proteins ALDOA and ALDH3A1 were critical signaling components involved in the mechanism by which irradiated lung cancer cells promoted the migration of recipient cells by accelerating glycolysis.^427^ Valya et al.^428^ reported that irradiation induced the export of miR‐603 via EVs, which promoted the CSC state and acquired radioresistance in glioblastomas. Similarly, mi‐208a can be transported by EVs and affect the proliferation and radiosensitivity of lung cancer cells by targeting p21.^429^

Studies have demonstrated that cargos of EVs or drugs targeting EVs have a synergistic effect with traditional antitumor therapy through enhancing immune cell activation and inducing cancer cell death.^430^ Botulinum neurotoxin type A, which has been reported to have antitumor effects, augments the efficacy of PD‐1 inhibitors on MC38 tumors through reducing serum exosome levels and improving the population of tumor‐infiltrating CD4+ and CD8+ T lymphocytes.^431^ The United States Food and Drug Administration‐approved antagonist macitentan, a dual endothelin receptor A/B (ETA/ETB) antagonist used to treat pulmonary arterial hypertension, significantly increased antitumor efficacy by inhibiting exosome secretion and increasing CD8+ T‐cell quantity and activity while lowering Treg numbers in tumors and draining lymph nodes when combined with an anti‐PD‐L1 drug.^322^ One study reported that sulfisoxazole‐mediated inhibition of tumor‐derived exosomal PD‐L1 has the capacity to impede immune evasion. Combining sulfisoxazole with anti‐PD‐1 therapy in animal models led to a significant decrease in exosomal PD‐L1 levels in the blood and the activation of CD8+ cytotoxic T cells.^432^ PS, which is expressed on the surface of exosomes derived from human TMEs, has been causally associated with T‐cell immunosuppression. Richard et al.^433^ synthesized a novel compound consisting of (ZnDPA)6‐DP‐15K designated as ExoBlock, which is a multivalent PS binder that substantially inhibits the immunosuppressive activity of exosomes, leading to an increase in both the quantity and functionality of CD4+ and CD8+ T cells in ovarian tumors and melanoma. The nonsteroidal anti‐inflammatory drug sulindac has been demonstrated to impede the development and progression of CRC with the mechanism of a reduction in exosomal PD‐L1. As a result, sulindac can potentially enhance the overall efficacy of anti‐PD‐L1 therapy.^434^ However, to confirm the efficacy of these pharmaceuticals in combination with PD‐1 inhibitors, additional clinical trials are needed.

## 6 CLINICAL APPLICATIONS OF EVs IN CANCER MANAGEMENT

### 6.1 EV‐based liquid biopsies for personalized cancer treatment

A major advancement in contemporary medicine is personalized cancer therapy, which entails creating treatment plans based on the features that make each patient's cancer distinct.^435^ Imatinib is a medication that may be the embodiment of customized treatments.^436^ The treatment for chronic myelogenous leukemia (CML), imatinib, selectively deactivates a mutant tyrosine kinase called BCR–ABL, which is encoded by the fusion of two genes.^437^ This genetic construct causes uncontrollable growth and malignant transformation in cells as a result of an aberrant chromosomal translocation. Therefore, a medication that targets this gene's product ought to destroy leukemia cells only while sparing healthy cells. That's exactly what it accomplishes, demonstrating remarkable efficacy and minimal harm. Another excellent example of personalized cancer treatment is using immunotherapies, including PD‐1 blocking antibodies, in a customized manner, giving pembrolizumab to patients whose tumors express PD‐L1 at measurable levels.^438^, ^439^

EVs have been the subject of a significant amount of effort to convert them into cancer biomarkers. Nevertheless, the recent focus is on the potential of these biomarkers to transform the field of personalized treatment. There are two primary applications for EVs in adaptive medicine: adjusting the initial treatment plan as needed during therapy using routine liquid samples to track a patient's reaction; making the best clinical decisions for each patient.^440^ Several studies demonstrated that specific drugs have the potential to cause cancer cells to alter their EV emission characteristics following CTX therapy, which could indicate drug‐related therapeutic stress.^441^ For instance, EGFR, p‐EGFR, and gDNA‐containing exosome‐like EVs were released in large quantities when treated with the EGFR inhibitor CTX.^442^ As a result, it is possible to characterize and use these EV emission profiles to assess the effectiveness of specific treatments in individuals.

### 6.2 EV‐based vaccines and immunotherapeutic strategies

Therapeutic cancer vaccines have been investigated as a potential advanced weapon to provide clinical advantages for cancer patients.^443^ Dex is thought to be a viable approach for DC‐based immunotherapy because it maintains the essential immunostimulatory characteristics of DCs.^444^ TDEVs produce tumor‐specific antigens that may also be recognized and processed by DCs, leading to the activation of DCs and the stimulation of antigen‐specific CD8+ T lymphocytes, which in turn exhibit antitumor effects. Following Dex treatment, the number of PD‐1+ CD8+ T cells significantly increased, suggesting that PD‐1 inhibitors combined with Dex may have combined antitumor effects.^445^ As a potential antitumor vaccine, the human endogenous MUC1 protein has been utilized to conjugate with Dex. The antibodies induced by this vaccine exhibited robust affinity toward tumor cells expressing MUC1 and elicited antigen‐specific CTLs and robust antibody response, leading to tumor eradication.^446^ In contrast, immature DCs promote inflammation and tumor progression because of their inability to stimulate cytotoxic T‐cell responses. In Ewing sarcoma, EVs reduced the expression of costimulatory molecules associated with DC maturation (CD74, CD86, and HLA‐DR) while also inducing cytokine release, including IL‐6, IL‐8, and TNF of CD33+ myeloid cells and CD14+ monocytes. EVs inhibited the proliferation of CD4+ and CD8+ T cells and IFN‐γ release. As a result, antigen‐presenting cell differentiation and function may be compromised by EVs, thereby diminishing adaptive immunity.^447^ The advancement of Dex therapy is confronted with a formidable obstacle. To facilitate the extensive application of Dex, it is imperative to devise an advanced technique for its synthesis that incorporates a greater quantity of pure exosomes. Dex has numerous complex mechanisms and effects on the immune system that have not been fully elucidated. Because Dex vaccine research is still in its infancy, additional investigations are necessary to improve the understanding and application of Dex.^448^

Mature Dex (mDex) has been shown to ameliorate the immunosuppressive TME (Table 4). Mechanistically, mDex has been found to have a greater capacity to stimulate naïve DCs and T cells than exosomes isolated from immature DCs, as evidenced by their increased expression of MHC and costimulatory molecules. mDex in combination with PLX‐3397, a small molecule inhibitor of CSF‐1/CSF‐1R, enhanced CD8+ T‐cell infiltration and depleted TAMs and MDSCs in the TME of mouse melanoma model, resulting in prolonged survival and delayed tumor growth.^449^ It was suggested that immunotherapy based on mDex provides a therapeutic approach for the management of solid malignancies.

**TABLE 4:** An overview of clinical applications of extracellular vesicles in tumors.

Cargos	Tumors	Origin	Functions	Status (start time)	Type	ClinicalTrials.gov ID or references
MAGE tumor antigens	NSCLC	Dex vaccine	Safety, feasibility, and efficacy	Completed (2003)	Interventional	^450^
MAGE 3 peptides	Melanoma	Dex vaccine	Safety, feasibility, and efficacy	Completed (2003)	Interventional	^451^
MHC I and II tumor antigens	NSCLC	IFN‐γ‐Dex vaccine	Efficacy	Completed (2010)	Interventional	^452^
SART1 peptide	Esophageal cancer	Dex vaccine	Safety, feasibility, and efficacy	Completed (–)	Interventional	^453^
CEA, MHC molecules, HSPs	CRC	Ascite‐derived exosomes	Safety, feasibility, and efficacy	Completed (2006)	Interventional	^454^
Exosomes	Bladder cancer	Chimeric exosome vaccine	Safety, feasibility, and efficacy	Unknown (2022)	Interventional	NCT05559177
Thyroglobulin, galectin‐3, calprotectin A9	Follicular thyroid cancer	Urine	Early and preoperative diagnosis	Recruiting (2022)	Observational	NCT05463107
Integrins, matrix metalloproteinases	Colorectal cancer	Blood	Noninvasive prospective approach in the screening of protein markers for diagnostic and/or prognostic	Completed (2021)	Observational	NCT04394572
Proteins	Lung cancer	Blood	Early diagnosis	Unknown (2020)	Observational	NCT04529915
CD9+/CA9+ exosomes, CD9+/ VGEFR2+ exosomes, CD9+/CD63+/ CD81+/CA9+ exosomes	Clear cell renal cell carcinoma	Urine	Early diagnosis	Recruiting (2020)	Observational	NCT04053855
CD20, PD‐L1	Blood	Non‐Hodgkin B‐cell lymphomas	Therapeutic resistance	Recruiting (2019)	Interventional	NCT03985696
PD‐L1 mRNA	Blood	NSCLC	Consistency analysis of PD‐L1 in cancer tissue and plasma exosome	Completed (2016)	Interventional	NCT02890849
PD‐L1 mRNA	Blood	NSCLC	Consistency analysis of PD‐L1 expression level detected in cancer tissues and plasma before and after radiotherapy	Completed (2017)	Interventional	NCT02869685
PD‐L1 protein, exosomal LAG‐3	Blood	HCC	Predict the efficacy of immunotherapy	Recruiting (2023)	Observational	NCT05575622
PD‐L1, miRNA	Blood	NSCLC	Predict the efficacy of immunotherapy	Unknown (2020)	Interventional	NCT04427475
Proteins	Primary cell cultures	Oropharyngeal carcinoma	Screening modality	Recruiting (2015)	Observational	NCT02147418
lncRNA‐GC1	Blood	Gastric cancer	Potential biomarker for detection	Unknown (2022)	Observational	NCT05397548
sncRNAs	Urine	Bladder cancer	Diagnosis	Recruiting (2020)	Observational	NCT04155359
mRNA	Blood	Pancreatic cancer	Diagnostic and prognosis	Unknown (2018)	Observational	NCT03821909
miRNA, lncRNA	Blood	Ovarian cancer	Detection and prognosis	Unknown (2018)	Observational	NCT03738319
RNA	Blood	Osteosarcoma	Biomarkers for lung metastases	Completed (2017)	Observational	NCT03108677
lncRNA	Blood	Lung cancer	Diagnosis	Completed (2017)	Observational	NCT03830619
ncRNAs	Blood	CCA	Diagnostic and prognosis	Unknown (2017)	Observational	NCT03102268
RNA signature	Urine	Prostate cancer	Association with the presence of any Gleason grade	Completed (2014)	Observational	NCT02702856
Kras G12D siRNA	Mesenchymal stromal cell	Pancreatic cancer	The best dose and side effects	Recruiting (2021)	Interventional	NCT03608631
Curcumin	Plant	Colon cancer	Ability to deliver curcumin	Recruiting (2011)	Interventional	NCT01294072
Tumor antigen	Dendritic cell	NSCLC	Enhance NK cell antitumor immunity	Completed (2010)	Interventional	NCT01159288

*Data sources*: ClinicalTrials.gov.

Abbreviations: CCA, cholangiocarcinoma; CRC, colorectal cancer; Dex, dendritic cell‐derived exosomes; HCC, hepatocellular carcinoma; HSPs, heat shock proteins; MHC, major histocompatibility complex; NK, natural killer; NSCLC, non‐small cell lung cancer.

In addition to Dex, alternative mechanisms by which EVs induce an antitumor immune response have been demonstrated. Tumor‐derived circulating exosomal MHC‐I is crucial for immune system activation. Combining immune checkpoint inhibition with MHC‐I overexpression in exosomes allowed glioma cells to present antigens again and stimulated CD8+ T cells to mount a powerful antitumor immunological response.^455^ Shin et al.^456^ reported that EVs derived from CD4+ T cells enhance the proliferation and activity of CD8+ T cells, thereby augmenting their antitumor response. Emerging evidence has indicated that CD8+ T cells may also perform exosome delivery to recipient tumor cells, thereby inducing the release of specific cargoes, including mRNAs, miRNAs, proteins, and lipids to kill tumors.^457^ VδT cells are MHC‐unrestricted lytic innate‐like T cells. They have tremendous immunotherapeutic potential against malignancies. Vδ2 T cells can be found in the peripheral circulation and lymphoid tissues. A T‐cell‐mediated antitumor response was produced by Vδ2 T‐cell‐derived exosomes, which led to more effective suppression of EBV‐associated malignancies.^458^ CAR‐T cells are potentially effective new treatments for cancers, partially because of the EVs released by CAR‐T cells. Exosomes harboring CARs express a high concentration of cytotoxic biological molecules and prevent the growth of tumors.^459^ In addition, the M1 macrophage‐derived exosomes can reinforce the CTL response by acting as immune adjuvants.^460^ It has been demonstrated that inducing the active release of EVs by NK cells could be an effective therapeutic strategy. NK cells and NK‐derived EVs were significantly more abundant in NSCLC patients than in healthy donors. Additionally, the number of NK cells and the quantity of CTCs exhibited a negative correlation.^461^

### 6.3 Clinical trials utilizing EVs as therapeutic agents or biomarkers

Since EVs are rich in parent cell‐derived proteins, they provide a unique window into cancer processes. The capacity of EVs to deliver accurate and trustworthy information is what supports the increasing acknowledgment of their potential in therapeutic applications.^462^ Proteins enclosed within protective lipid bilayers make them more accurate diagnostic tools with a lower chance of false positives, which is especially helpful in situations involving heterogeneous cell populations such as tumors. The usefulness of proteins in EVs as biomarkers has already been shown in a few clinical studies, such as thyroglobulin in the urine of follicular thyroid cancer patients (NCT05463107) or integrins in CRC patient blood (NCT04394572) as shown in Table 4. Actually, among the numerous proteins, the identification of the most specific protein remains a critical issue that must be resolved in the future.

As we showed in the above in vitro and in vivo study, nucleic acids (mRNA, miRNA, lncRNA, circRNA) in EVs have the potential to function as biomarkers. Clinical trials for EVs as diagnosis and therapeutic agents are currently underway, and we have updated them in Table 4 to reflect their potential medicinal benefits. After being internalized by recipient cells, mRNA produced from EVs can be translated into proteins, which can control the biological activities of receptor cells in addition to serving as a signal carrier. New forms of RNA are constantly appearing, joining the already established mRNAs and ncRNAs.^463^ These include chimeric RNAs, tRNAs, and piRNAs, which are also explored in recent clinical trials (ChiCTR2000031507).

A phase I trial investigates the optimal dosage and side effects of exosomes from mesenchymal stromal cells carrying KrasG12D siRNA (iExosomes) for the treatment of patients with metastasic pancreatic cancer (NCT03608631). The purpose of another clinical trial is to determine whether plant exosomes can more efficiently transport curcumin, which has a potent antitumor growth inhibitory effect, to both normal colon tissue and colon cancers (NCT01294072). In phase II clinical research, Chaput et al.^452^ determined the clinical benefit of IFN‐γ‐Dex loaded with cancer antigens in patients with NSCLC that is incurable and does not exhibit tumor progression as maintenance immunotherapy following induction chemotherapy. Since the sources of EVs are diverse, they can be used as a variety of medication carriers. Examining the safety and effectiveness of EVs as medication delivery vehicles and investigating their possible therapeutic applications in a range of diseases are the goals of further clinical trials.

## 7 CHALLENGES AND FUTURE PERSPECTIVES

### 7.1 Standardization of EV isolation and characterization protocols

Numerous biofluids, including blood, urine, sweat, and milk, are frequently reported to contain EVs.^464^ However, protein complexes and biomacromolecules can make it difficult to separate and collect from these complex biofluids.^465^ Numerous methods have been developed according to density, including two widely used techniques: density gradient centrifugation and differential ultracentrifugation (Figure 4).^466^ Notably, there are certain restrictions associated with these methods. The structural integrity of exosomes may be compromised by the intense centrifugal forces during ultracentrifugation, which requires expensive apparatus and huge sample volumes. Compared with centrifugation, size‐based isolation such as sequential filtration and size‐dependent microfluidics has many benefits.^467^ Reducing the size of the device results in lower sample volume needs, higher throughput, and enhanced sensitivity. Because of its adaptability and label‐free ability to separate exosomes by size, the microfluidic method has great promise for clinical applications.^468^ Many researchers prefer polymers like polyethylene glycol (PEG)‐based methods for exosome separation recently.^469^ Sedimentation of EV products after incubation in a PEG solution is observed near the tube base after low‐speed centrifugation. Although this kind of coprecipitation technique provides a simple isolation process, its wider utility may be limited by its relatively poor throughput and the possibility of chemical contamination. Another technique known as immunoaffinity‐based capture (IAC) takes advantage of the binding affinity between the receptors and ligands interactions. As a result, exosomes can be specifically isolated from bodily fluids, thereby facilitating the use of IAC in isolating tumor‐specific exosomes for liquid biopsies and disease diagnostics.^470^ Studies have demonstrated that melanoma‐specific exosomes may be successfully separated and isolated with an efficiency of about 95% using the IAC‐based technique by recognizing melanoma‐associated antigens.^471^ Likewise, exosomes unique to AML were successfully extracted from cell culture supernatants using an anti‐CD34 antibody‐based IAC technique. Just 10 µL aliquots of the CD34 microbeads were able to capture all the exosomes present in 100–1000 µL of AML solution, demonstrating the remarkable effectiveness of microbeads used in this method.^472^ Although there are numerous benefits to using IAC for exosome separation, such as its high efficiency and purity, the method incurs substantial expenses due to the utilization of antibodies, and magnetic beads. In general, it is imperative to take into account the specific needs of the application in order to determine the most appropriate method for isolating exosomes from complex biofluids. In this manner, it can be guaranteed that the selected method will satisfy the appropriate criteria such as purity, compatibility, efficacy, and yield for subsequent analyses.

Further validation of characterization is necessary for extracted and purified exosomes, and it offers a valuable means of confirming the efficacy of their extraction process and supplying a solid foundation for their potential uses. The prevailing techniques employed to quantify exosomes include measuring the total protein and particle amount. In addition, qualitative characterization necessitates the use of imaging techniques and biophysical features.^473^ The surface morphology information can be shown by electron microscopy. The particle concentration and size distribution can be found by nanoparticle tracking analysis and dynamic light scattering, which measures the exosome size and potential distribution.^474^ Exosomal proteins can be analyzed using several techniques such as enzyme‐linked immunosorbent assays, chromatography, flow cytometry, protein blotting, and MS.^475^ Recently, there have been advancements in protein detection methods, including electrochemical detection, colorimetric detection, surface‐enhanced Raman scattering, fluorescence detection, surface plasmon resonance detection, and CRISPR/Cas detection.^303^ Moreover, MS methods are also used for the examination of exosomal lipids. In order to establish the nature of exosomes as enclosed vesicles with a lipid bilayer structure, it is necessary to analyze and identify at least one protein that binds to the membrane or lipids, as well as one protein existed in the cytoplasm. For the analysis of exosomal RNA and DNA, techniques such as digital PCR, real‐time fluorescence quantitative PCR, and NGS sequencing can be utilized.^476^ Similarly, novel techniques for detecting nucleic acids, including single vesicle analysis, CRISPR/Cas‐assisted detection and thermophoretic detection have recently been developed.^303^ As a result, advancements in exosome detection technologies have made them more accessible for diagnostic purposes. It is worth mentioning that exosomes can alter their physical or biological characteristics. To summarize, a thorough analysis of exosomes allows for the identification of their characteristics from several perspectives, hence offering substantial evidence for their future use.

### 7.2 Safety considerations and regulatory challenges for EV‐based therapies

Numerous technical obstacles that impede the development of EV‐based therapies have appeared. As for the isolation methods, newer techniques like size exclusion chromatography and tangential flow filtration have the potential to handle higher amounts of EVs.^477^ It is also imperative to figure out EVs populations with inherent heterogeneity which could exhibit maximum functional activity by enhancing isolation procedures and developing the potency assays capability. Further complexity arises when production is highly needed for therapeutic applications including bioreactors and media supplements (e.g., fetal bovine serum containing exosomes). To take safety into consideration, using suitable preclinical models and careful cargo selection will be necessary to determine the safest cell source for exosome isolation in order to reduce immunogenicity and undesired cargo.^47^

A fuller comprehension of the delivery pathways is necessary for attempts to direct EVs to certain areas for therapeutic intervention. In recent breakthroughs, targeting specificity has been demonstrated in EV surfaces with the use of RNA aptamers or nanobody fragments.^478^ EVs have demonstrated potential for increasing tropism toward specific cells or organs through engineering; however, the production of EVs with therapeutic potential remains a challenge.

As widely known, EV components, encompassing glycans, proteins, nucleic acids, or lipids, exhibit great promise in cancer treatment. However, there is a long way to go to translate these findings into therapeutic applications. As a result, it is crucial to standardize clinically appropriate EV preparations, especially when it comes to figuring out how EVs work, how much is a good dose, and how they work.

### 7.3 Challenges for harnessing EVs for precision medicine and targeted drug delivery

EVs have shown great promise recently as drug carriers for targeted tumor therapy, but their practical applicability is still limited by a number of issues. The safety of EVs should be taken into account when they are employed as delivery vehicles due to their biological activity. TDEVs contain constituents that stimulate tumor proliferation, invasion, and metastasis. As such, there are potential dangers associated with hastening tumor progression.^394^ As a result, additional clinical research on EV treatment is required, with an emphasis on the toxicological features and safety of clinical trials. The content and properties of EVs are influenced by both upstream and downstream alterations of the source cells, leading to a range of therapeutic outcomes. Consequently, minimizing batch variation while preserving stable EV structure and characteristics may be achieved by managing the stability of the culture environment and creating efficient EV processing procedures.^479^ A solution to this issue might be the creation of more effective techniques for EV extraction and purification or the investigation of novel EV sources. The mechanisms behind the selective cellular uptake of EVs and the patterns of their intracellular distribution remain poorly understood, potentially resulting in off‐target occurrence. When executing membrane‐disrupting manipulations, such as cargo delivery to EVs, there is a danger of changing the orientation of membrane proteins. This could lead to identification by the immune system and subsequent negative reactions. The process of isolating, loading drugs, and modifying delivery methods is converted into the release of drugs at the location of the lesion, thereby eliminating the potential risk of altering the features and characteristics of EVs efficiently.^480^

EVs are difficult and expensive to mass‐produce using current isolation methods as mentioned above. Moreover, it is yet unknown what the requirements are for production, loading efficiency, purification, storage, dosage, and clinical application.^481^ Despite promising potential, only a small number of EV‐based treatments have made it into clinical trials due to the disparity between laboratory and clinical applications.^482^ Thus, more attention should be paid to safety and toxicological profiles, more effective isolation methods and production, specific drug delivery, optimizing modification techniques, standardizing from production to application, and comprehensive mechanisms of intracellular distribution and selective cellular uptake in EV‐based drug delivery research.

## 8 CONCLUSIONS AND PERSPECTIVES

Recent results indicate that EVs and their cargo play a pivotal role in the various clinical diseases, including cancer,^483^ cardiovascular diseases,^484^ neurological diseases,^485^ and infectious diseases.^486^ We mainly elucidated the crucial function of EVs as a signaling hub in the TME and highlighted numerous essential processes of cancer progression, such as oncogenic signal transfer, angiogenesis, metabolic reprogramming and immunosuppressive microenvironment remodeling, all of which significantly facilitate drug resistance and tumor progression. Encapsulated within the lipid bilayer of EVs, extensive cargo is shielded from enzyme degradation, maintaining the original structure and functionality and serving as excellent drug carriers. Since the EV content changes according to the parent cell, EVs can serve as particular biomarkers that reveal details about the molecular and genetic heterogeneity of tumors. Therefore, developing EVs as drug vehicles and therapeutic targets is a method with great potential. Ongoing research continues to elucidate these roles and applications in various cancer types including as biomarkers, therapeutic agents, drug delivery vehicles, cancer vaccines, and other individualized cancer management strategies. Despite recent progress in the study of EVs in cancer, inquiries regarding their clinical use remain untouched.

An immense number of studies have recently explored the roles of EVs in tumor progression. Our emphasis was on the diverse effects of EVs that come from various sources, transport different loads, and influence various TME components. However, we are still in the early stages of understanding EVs in cancer. EVs possess a heterogeneous nature that renders them capable of facilitating tumor growth and proliferation under specific circumstances. Hence, it is critical to continue diligently investigating EVs and their supplementary biological attributes to harness their biological capabilities for the advancement of an innovative line of cancer therapies.

However, there are still numerous obstacles facing EV‐based cancer treatment techniques such as isolation and purification as we mentioned. Apart from technical problems, it is not yet known which genes control the loading and secretion of EVs. More research is required to determine which EVs can precisely represent tumor diagnosis and treatment response, as well as which ligands and receptors precisely guide EVs to particular receptor cells. EVs have been the subject of numerous clinical trials for tumor therapy to date. Moreover, to advance the investigation of EVs, larger‐scale, multicenter, and longer‐lasting clinical trials are needed.

## AUTHOR CONTRIBUTIONS

*Conceptualization*: C. L. and Y. Z. *Manuscript writing*: Y. M. and X. Z. *Manuscript review and editing*: C. L. and Y. Z. All authors have read and agreed to the published version of the manuscript.

## CONFLICT OF INTEREST STATEMENT

The authors declare no conflict of interest.

## ETHICS STATEMENT

Not applicable.

## Data Availability

Not applicable.
